# Oxidative Stress Biomarkers in Fish Exposed to Environmental Concentrations of Pharmaceutical Pollutants: A Review

**DOI:** 10.3390/biology14050472

**Published:** 2025-04-25

**Authors:** Lăcrămioara Grădinariu, Mirela Crețu, Camelia Vizireanu, Lorena Dediu

**Affiliations:** 1 Faculty of Medicine and Pharmacy, “Dunărea de Jos” University of Galaţi, 35 A.I. Cuza Str., 800010 Galaţi, Romania; lacramioara.gradinariu@ugal.ro; 2 Faculty of Cross Border, “Dunărea de Jos” University of Galați, Domnească Street, No. 47, 800008 Galați, Romania; 3 Romanian Center for Modelling od Recirculating Aquaculture System, “Dunărea de Jos” University of Galaţi, Dr. Alexandru Carnabel No. 61, 800201 Galați, Romania; camelia.vizireanu@ugal.ro; 4 Faculty of Food Science and Engineering, “Dunărea de Jos” University of Galați, Domnească Street, No. 111, 800008 Galați, Romania

**Keywords:** fish, environmental concentrations, pharmaceutical pollutants, antioxidant defense, biomarkers of oxidative stress

## Abstract

Pharmaceutical residues are a result of human activities and are increasingly recognized as environmental contaminants that pose significant risks to aquatic ecosystems. There are many well-known pathways (agricultural runoff, veterinary use, human excretion, etc.) for the entry of these pharmaceuticals into the aquatic environment, and among them, the inability to remove these biologically active compounds from wastewater treatment plant (WWTP) effluents is becoming increasingly significant in the context of societal evolution. Once introduced, pharmaceuticals persist at low concentrations, exerting sub-lethal effects that disrupt the physiological processes of aquatic organisms. Among these effects, oxidative stress (OS) has gained attention as a key mechanism that is induced by pharmaceutical toxicity, serving as a sentinel indicator of homeostatic disturbance. Thus, studying OS biomarkers in fish is crucial for understanding the extent of pharmaceutical pollution, as these biomarkers provide early warning signals of environmental stress and help assess sub-lethal impacts on aquatic organisms. Their application, correlated with other eco-physiological investigations, can improve ecological risk assessments. In this context, this review explores the role of OS biomarkers by assessing the effects of pharmaceutical contaminants on fish. It highlights the utility and limitations of these biomarkers for environmental monitoring, while also identifying key research gaps—particularly regarding long-term ecological consequences.

## Simple Summary

Pharmaceutical pollution has become a great concern as it has significant consequences on the aquatic environment, particularly on the living organisms within it. In this context, this review provides an extensive analysis of the impact of waterborne pharmaceuticals on fish, focusing on oxidative stress (OS) biomarkers. It offers a systematic evaluation of the reactions of fish to environmental concentrations of the most common pharmaceuticals found in aquatic environments, as documented in both in situ and ex situ laboratory exposure studies. Key oxidative stress biomarkers, such as catalase (CAT), superoxide dismutase (SOD), glutathione peroxidase (GPx), glutathione-S-transferase (GST), and glutathione reductase (GRed), are described and discussed, highlighting their significance in the assessment of the biochemical and physiological responses of fish to pharmaceutical contaminants. Furthermore, this review examines the effects of specific pharmaceuticals on OS across various fish species, highlighting differences in species-specific sensitivities.

## 1. Introduction

### 1.1. The Sources and Types of Human Pharmaceutical Active Compounds Within the Aquatic Environment

Human activities, including industrial production and agriculture, have significantly contributed to environmental pollution, introducing a range of contaminants such as plastics [1], pharmaceutically active compounds (PhACs) [2], pesticides [3], and heavy metals [4]. These pollutants accumulate in aquatic and terrestrial ecosystems, leading to ecological imbalances and posing significant threats to biodiversity and ecosystem health.

The global rise in pharmaceutical consumption has significantly contributed to this issue. Statistics from the European Union (EU) reported the use of over 3000 medicinal substances for human medicine [5,6]. The 2023 public report published by the IQVIA Institute for Human Data Science predicted an increase in per capita pharmaceutical consumption across most regions, amplifying further concerns about the environmental impacts of these compounds [7].

Many pharmaceuticals and their byproducts are not completely metabolized by humans and animals, leading to their presence in wastewater [8]. The improper disposal of expired or unused medications and drug residues from human and animal excretion are important sources of wastewater pharmaceutical pollution [9]. These compounds enter sewage systems and are only partially eliminated through WWTPs in natural aquatic ecosystems due to the inefficiency in treating such substances. Despite advancements in water treatment technologies, pharmaceutical residues continuously accumulate in rivers, lakes, and groundwater [10]. Furthermore, PhACs can infiltrate soil and groundwater through agricultural practices, such as the use of animal manure and wastewater sludge as fertilizers [11]. Other notable sources of pollution are industrial discharging, agricultural runoff, and waste production from pharmaceutical facilities [12,13,14].

Among the most detected classes of PhACs in aquatic environments (Table 1) are antibiotics, analgesics, antidepressants, anticonvulsants, beta-blockers, hormones, and antihistamines. Although these substances are found in water at low concentrations (ng/L to µg/L), the ecological consequences could be significant.

Once within aquatic ecosystems, PhACs can disrupt physiological processes in aquatic organisms, including fish. These pollutants can significantly impact fish physiological homeostasis [29], hormone regulation [30], reproduction [31], immune function [32], and OS responses [33,34,35]. Moreover, long-term exposure to pharmaceutical pollutants has also been linked to antimicrobial resistance [36], posing a significant environmental and human health threat. Despite growing awareness, the research assessing the impacts of pharmaceutical pollution on fish remains limited, hindering our understanding of how these pollutants affect aquatic ecosystems, mainly because there is a lack of information regarding long-term ecological consequences and how these pollutants influence species resilience and overall biodiversity. The contamination of aquatic environments with PhACs has become a primary worldwide concern, leading to scientific and government efforts to mitigate their environmental impact.

Recognizing the urgency of this issue, the European Commission (EC) has implemented regulatory actions under the Water Framework Directive (WFD, Directive 2000/60/EC) to assess and manage pharmaceutical pollution in surface waters. As part of these efforts, the Watch List Mechanism was introduced to gather high-quality monitoring data on emerging contaminants and determine whether specific substances requiring regulatory limits should be classified as priority substances [37]. In 2013, the European Commission identified three pharmaceutical compounds—17-alpha-ethinylestradiol (EE2), 17-beta-estradiol (E2), and diclofenac (DCF)—as substances of particular concern, adding them to the first EU Watch List [37]. These substances were selected based on their high toxicity, their persistence in aquatic environments, and their potential endocrine-disrupting effects [38]. This initiative aimed to collect sufficient environmental data to evaluate whether regulatory action, such as classification as priority hazardous substances, was necessary. For example, in 2018, diclofenac (DCF) was removed from the Watch List due to the availability of sufficient high-quality monitoring data [39] and due to its low bioaccumulation potential in aquatic ecosystems [39]. Indeed, several studies indicated that the environmental concentrations of DCF were generally below thresholds of concern for both ecological and human health risks [40]. However, subsequent studies have shown that DCF can cause acute toxicity, OS, organ lesions, and gene damage in aquatic animals [41]. The two endocrine-disrupting estrogens, EE2 and E2, remained on the list due to their potent biological activity, even at extremely low concentrations [42]. At the same time, in 2020, concerns regarding pharmaceutical pollution led to the inclusion of five additional compounds in the EU Watch List—amoxicillin, ciprofloxacin, sulfamethoxazole, trimethoprim, and venlafaxine [43]. In 2022, ofloxacin was added to the list, reflecting ongoing efforts to track and regulate emerging water pollutants [44]. All these regulatory actions demonstrate a growing recognition of the risks posed by pharmaceutical pollution to aquatic ecosystems as new pharmaceuticals are routinely assessed.

In this context, the present study plays a crucial role in advancing knowledge on the environmental impact of pharmaceutical pollution by synthesizing and analyzing findings from the scientific community. By focusing on pharmaceutical compounds with high incidence in aquatic environments, this research highlights their potential to induce oxidative stress in various fish species, a key biomarker of physiological and cellular disturbances. Therefore, this study contributes to the growing body of evidence necessary for regulatory decisions and the development of mitigation strategies to reduce pharmaceutical contamination and safeguard aquatic life.

### 1.2. Oxidative Stress (OS) and Antioxidant Defenses

Fish are exposed to PhACs through two main pathways: absorption from the surrounding water and the ingestion of the contaminated food [45]. When accumulation exceeds elimination, bioaccumulation occurs [46]. PhACs can then be distributed through the bloodstream, accumulate in specific tissues, and interfere with cellular processes like enzyme activity, hormonal regulation, and OS, resulting in the distribution of these chemicals to different organs [47,48]. For example, endocrine-disrupting chemicals such as hormones induce feminization in fish [49], alter their reproductive system, cause abnormal gonadal development [50], and influence behavior [51,52]. Nonsteroidal anti-inflammatory drugs (NSAIDs) also cause nephrotoxicity and impair normal physiological functions, potentially compromising the adaptive immune systems of fish [53]. Antidepressants may impair brain function, which leads to changes in behavior [54] and stress reactions, which in turn endanger the survival and welfare of the fish [55]. In addition, the bioaccumulation of PhACs may lead to OS, causing cellular damage in tissues such as the liver, gills, and kidneys [56] and disrupting key processes like osmoregulation and respiration [57].

To mitigate toxicity, fish employ biotransformation mechanisms [58] as a coping strategy. This key process involves two stages: Phase I reactions, catalyzed by cytochrome P450 (CYP450) enzymes, introduce polar functional groups to contaminants. If these modified compounds remain insufficiently water-soluble, Phase II reactions conjugate them with molecules such as glucuronic acid, sulfate, or glutathione (GSH), making them more water-soluble and thus enhancing their excretion from the body. However, incomplete metabolism or the formation of reactive intermediates, such as reactive oxygen species (ROS), can trigger OS, a key toxicity pathway [59,60].

OS occurs when ROS overwhelm antioxidant defenses, resulting in cell damage [61]. Free radicals, such as hydrogen peroxide (H_2_O_2_), hydroxyl radicals (OH^−^), and superoxide anion (O_2_^−^), are naturally occurring byproducts of cellular metabolism and immunological responses [62]. However, excessive ROS levels cause oxidative damage to proteins, lipids, DNA, and RNA. Free radicals can induce cellular damage, mainly when antioxidant protection mechanisms are compromised. While ROS, comprising loose radicals and free radicals like H_2_O_2_, are usually produced during regular cell features, their immoderate accumulation results in OS, which is responsible for numerous diseases and aging. These compounds further propagate oxidative damage, making them essential biomarkers for assessing OS in fish organisms. Lipid peroxidation (LPO) occurs when free radicals attack lipids in cell membranes and is one of the primary consequences of physiological changes during OS. ROS attack polyunsaturated fatty acids in cell membranes, which leads to structural instability and functional impairment [63]. The process also generates toxic byproducts such as malondialdehyde (MDA) and 4-hydroxynonenal (4-HNE) [64,65]

Protein oxidation or protein oxidative modifications occur when ROS modify proteins, altering structure and functionality [66]. Modifications impair enzymatic activity, disrupt cellular signaling, and hinder normal cell function. As a result, protein oxidation plays a significant role in the decline of cellular processes and can contribute to disease development [62]. Damage to DNA and RNA is among the most critical consequences of OS, as they impact the long-term viability of cells and tissues [67,68,69,70]. DNA damage involves the oxidation of genetic material, leading to strand breaks and base modifications which can result in mutations, genomic instability, and carcinogenesis [71,72,73]. Several studies have proved that fish exposure to pollutants causes cancer in different organs [74,75,76,77]. ROS production disrupts molecular stability, impairs protein synthesis, and alters cellular signaling pathways in RNA. OS can also damage RNA molecules directly, leading to structural modifications, strand breaks, and changed nucleotide integrity [78]. Prolonged exposure to ROS increases mutation rates and inhibits protein synthesis, likely due to impaired ribosome function and enhanced mRNA degradation. This genomic and transcriptomic damage disrupts normal cellular functions and contributes to various diseases and dysfunctions [79,80,81].

Given their sensitivity to environmental pollutants, OS biomarkers serve as early indicators of water contamination, allowing researchers to detect sublethal effects in fish before visible physiological impairments such as reduced growth, decreased reproductive success, or behavioral changes. Therefore, researchers can evaluate the significance of pollution levels by monitoring biomarker responses and, based on the results, develop mitigation strategies to protect aquatic ecosystems. Enzymatic biomarkers (Table 2) and non-enzymatic antioxidants (e.g., vitamins, thioredoxin, and carotenoids) are involved in managing OS in fish [80,82], providing valuable information about how pollutants induce cellular stress and disrupt normal cellular functions.

Therefore, studying OS biomarkers is a valuable approach in ecotoxicological risk assessment, as these biomarkers reflect the molecular responses of organisms to contaminant-induced toxicity [62,89,90]. By analyzing specific OS markers, researchers can understand how pollutants affect cellular function and overall organism health.

In this context, this review aims to examine the responses of OS biomarkers in fish exposed to environmentally relevant concentrations of pharmaceuticals, highlighting their relevance in advancing ecotoxicological risk assessments and shaping regulatory frameworks.

## 2. Methodology

We conducted a systematic review to assess the effects of concentrations of key pharmaceutical pollutants on the induction of OS in fish. In this regard, only studies that investigated these effects under controlled laboratory conditions were identified to highlight the effects of each category of pharmaceutical pollutants separately. Relevant articles were retrieved from the Web of Science database, using keywords such as “oxidative stress in fish” and “pharmaceutical”.

The search was limited to peer-reviewed articles, focusing only on experimental studies. Our research identified 366 eligible articles; however, only 358 were ultimately selected for analysis. Furthermore, only 193 studies were used in the review, mainly because we wanted to focus on the most relevant and valid studies for research.

The main criteria for selecting articles were as follows:

(1) excluding studies involving other animal species;

(2) excluding studies lacking quantitative data on oxidative stress;

(3) keeping only studies published between 2004 and 2024 since research on OS in fish has significantly advanced in the past two decades due to improved analytical techniques and a growing awareness of environmental contaminants’ impact on aquatic organisms;

(4) the quality of the data and their relevance to the paper’s main theme.

## 3. Results and Discussion

### 3.1. The Influence of Non-Steroidal Anti-Inflammatory Drugs (NSAIDs) on Oxidative Stress in Fish

Non-steroidal anti-inflammatory drugs (NSAIDs) are frequently detected in aquatic ecosystems. Although they have therapeutic benefits on human health, NSAIDs raise environmental issues due to their potential ecotoxicity, despite their therapeutic benefits. Among them, diclofenac (DCF), ibuprofen (IBU), paracetamol (APAP), and aspirin (ASA) are commonly detected in aquatic environments [21,91]. The exposure of fish to NSAIDs has been proven to disrupt the activity of key antioxidant enzymes, leading to increased LPO and OS. Alterations from LPO and OS contribute to cellular damage and impair physiological homeostasis by disrupting membrane integrity, leading to increased permeability, enzyme dysfunction, and impaired ion balance. Additionally, oxidative damage to proteins and DNA can impair metabolic activity, cause immune suppression, and reduce growth and reproductive performance in fish [92].

NSAIDs exert their primary effect by inhibiting cyclooxygenase (COX) enzymes, specifically COX-1 and COX-2, which are essential for the biosynthesis of eicosanoids, signaling molecules that regulate inflammation, immune responses, and other vital physiological functions [66]. Notably, NSAID metabolites are often more toxic than their parent compounds, further exacerbating their impact on fish [93]. These metabolites induce OS, disrupt mitochondrial function, and interfere with normal biochemical pathways. Additionally, NSAIDs may interfere with endocrine signaling, affecting stress regulation, growth, and fish reproduction. Despite growing evidence of NSAID-induced toxicity in fish, the precise molecular and cellular mechanisms underlying these effects are not entirely understood [94]. This is due to the involvement of multiple pathways, including OS, immune suppression, mitochondrial dysfunction, and endocrine disruption. Additionally, species-specific responses, the role of toxic metabolites, and the long-term effects of low-dose exposure are not fully elucidated.

#### 3.1.1. The Influence of Diclofenac (DCF) on Oxidative Stress in Various Fish Species

Among the NSAIDs studied, DCF is a significant pollutant in aquatic ecosystems. However, the potential for DCF to undergo biotransformation in aquatic organisms after absorption remains poorly explored. Biotransformation is the process through which organisms alter the chemical structure of a compound, such as DCF, by biochemical reactions, mainly in the liver or in analogous organs. In fish, this involves Phase I cytochrome P450 enzymes, which oxidatively modify the compound, and Phase II glucuronyltransferases, which conjugate the modified compound to enhance its water solubility and excretion [95]. While detoxification is nevertheless able to perform beneficial functions, the process sometimes produces metabolites that have a more significant toxicity than that of the parent compound, thus exacerbating environmental and ecological hazards. In mammals, the primary phase I metabolites of DCF are 4′-hydroxy diclofenac (4′-OH-DCF) and 5′-hydroxy diclofenac (5′-OH-DCF). These metabolites undergo further oxidation, forming benzoquinone imine, a compound known for its toxicity to fish [96]. This biotransformation process induces the generation of ROS and leads to interactions with sulfhydryl groups in the cytosol, as well as in enzymes and membrane proteins containing these groups [97,98].

DCF has been detected in varying concentrations in water bodies worldwide, and it is recognized as a pseudo-persistent micropollutant (Table 3). In Romania, Chițescu et al. [18,99] investigated DCF levels in the Argeș River, reporting concentrations between 166 and 252 ng/L. Hallare et al. [100] found that in Central European surface waters, DCF concentrations can reach up to 0.54 μg/L. Additionally, metabolites of DCF, including 4-hydroxy diclofenac and 5-hydroxy diclofenac, have been detected in sewage treatment plant effluents in Germany at concentrations ranging from 0.07 to 0.42 µg/L [101].

Consequently, DCF and its environmental degradation products may impose significant ecological risks. Exposure to these compounds enhances ROS production and impairs the antioxidant defense mechanisms of fish, even at ecological concentrations (ng/L).

The exposure of male tiger fish (*Hoplies malabaricus*) to DCF through intraperitoneal inoculation at doses of 0.2, 2, and 20 μg/kg significantly increased antioxidant responses in the liver, suggesting the generation of free radicals. This indicates that the liver activates antioxidant defenses to prevent possible cellular damage when DCF causes oxidative stress. In particular, GPx activity was elevated at all tested doses, GSH levels increased at 20 μg/kg, and SOD activity was elevated at doses of 2 and 20 μg/kg. Furthermore, DCF caused hepatic LPO in every exposed group, suggesting the occurrence of OS. Also, DCF decreased the liver’s GST activity, indicating that biotransformation processes were inhibited (Table 4). By transforming superoxide radicals (O_2_^−^) into hydrogen peroxide (H_2_O_2_), whic is subsequently further neutralized by GPx and CAT to reduce oxidative damage, these results highlight the function of SOD as the first line of antioxidant defense [107].

According to Islas Flores et al., DCF biotransformation in the liver, hepatotoxicity, and hepatocyte apoptosis [114] primarily occur through the generation of ROS. Several authors [115,116] suggest that mitochondria are the primary site of ROS production following DCF exposure. DCF disrupts mitochondrial function by impairing adenosine triphosphate (ATP) synthesis and inhibiting key complexes of the electron transport chain. This mitochondrial dysfunction exacerbates ROS generation, further compromising cellular homeostasis and ultimately triggering hepatocyte apoptosis. Justi et al.’s meta-analytic research [117] highlights the significant contribution of GPx in reducing ROS accumulation and recognizes it as the most sensitive biomarker to NSAID-induced OS. Consistent with this finding, research on *Danio rerio* embryos and larvae exposed to DCF (0, 0.5, 5, 50, and 500 μg/L for 96 h) indicated increased GPx and CAT activity alongside decreased GST activity [108]. These biochemical alterations suggest an adaptive cellular response to OS. Elevated CAT activity reflects a defensive mechanism against increased hydrogen peroxide (H_2_O_2_) levels, whereas enhanced GPx activity indicates a compensatory reaction to excess ROS. In contrast, Diniz et al. [118] predicted that GST inhibition might be attributable to oxidative damage.

GST inactivation indicates enzyme sensitivity to OS and is supported by CAT and GPx overexpression caused by DCF exposure, suggesting ROS overgeneration. Furthermore, the increase in GPx activity implies enhanced glutathione (GSH) oxidation, highlighting its crucial role in counteracting oxidative damage. These findings underline the necessity of activating anti-oxidative mechanisms to normalize cellular homeostasis, further verifying the interrelation of OS and DCF-mediated mitochondrial dysfunction.

Likewise, Eze et al. [109] analyzed the effects of DCF exposure on Nile tilapia (*Oreochromis niloticus*). The experiment consisted of the exposure of fish to three sub-lethal concentrations of DCF (250, 320, and 480 µg/L) for 28 days and a 7-day recovery period. The results showed that DCF exposure induced OS in a dose- and time-dependent manner.

The significant increase in LPO with DCF exposure underscores the drug’s capacity to enhance ROS production, which can lead to DNA damage, protein oxidation, and physiological disturbances. The observed decreased SOD and CAT activities were consistent with increased LPO levels, which indicates OS suffered by the fish due to lack of antioxidant enzyme inhibition. Moreover, GRed activities and GPx increases may represent a compensatory response to OS. GPx plays a crucial role in reducing H_2_O_2_ and lipid peroxides, thereby preventing the formation of radical intermediates through oxygen reduction mechanisms [113]. Increasing GPx activity supports the idea of mitigating excessive ROS production [113]. In this context, an increase in GPx levels emphasizes the adaptive response to OS caused by DCF exposure. This adaptation, however, appears insufficient to fully counteract the oxidative damage, as evidenced by the persistent elevation of LPO. Interestingly, the increase in GSH activity indicates an upregulation of the antioxidant defense system, although not enough to prevent the adverse effects of prolonged diclofenac exposure. Generally, GSH is critical in maintaining immune system function and exhibits antioxidative, integrative, and detoxifying effects. The slight recovery of *Orechromis niloticus*, noted after a 7-day withdrawal period, suggests that the fish has some ability to recover from OS. Nonetheless, the authors reported incomplete recovery, particularly for SOD and CAT activities. This partial recovery indicates that DCF has long-standing environmental consequences, especially from chronic exposure (Table 4).

In contrast to the previous research, some authors suggest that DCF may protect some fish-specific physiological or biochemical processes. For example, Guiloski et al. [110] noted that the application of DCF at environmental doses (0, 2, 20 μg/L) in the fish species *Rhamdia quelen* decreased the activities of some antioxidant enzymes like SOD and CAT, alongside increases in GSH concentrations and GST activity (Table 4). These changes, which are a key part of the lipid membrane damage antioxidant defense system, most likely explain the decline in lipid membrane damage. The study illustrates DCF’s protective impact against LPO and helps explain the lack of protein carbonylation and DNA damage. A particularly significant finding was the reduction in LPO at concentrations of 0.2 and 20 µg/L of DCF, where the authors reported no evidence of DNA damage. Additionally, CAT activity only decreased in the 2 µg/L exposure group, and the effect was not concentration-dependent. The observed increase in GSH levels and GST activity likely played a critical role in preventing DNA damage in the blood and liver of *Rhamdia quelen* (Table 4).

Juveniles *Rhamdia quelen* exposed to DCF for 96 h at 0.2, 2, and 20 μg/L showed notable physiological changes. In the kidney, SOD activity was enhanced in all concentrations, suggesting some disruptions in H_2_O_2_ solubilization, though no DNA damage or lipid peroxidation was observed. These findings underscore the risks associated with even low levels and short durations of DCF exposure. However, the authors reported no evidence of OS in the liver [111]. These findings support the assertion of Petersen et al. [119], who stated that even low doses of DCF exposure can protect cells from OS. Moreover, Feito et al. [120] also reported the decrease in LPO after exposure to 0.03 µg/L DCF for 90 min in zebrafish embryos. Similarly, Praskova et al. [121] observed a decrease in LPO levels in 20-day-old zebrafish exposed to 0.02 µg/L DCF, an environmental concentration of DCF found in rivers of the Czech Republic, over 28 days. However, the authors reported no changes in GST and GRed enzyme activity. The reduction in LPO observed in these studies can be associated with a rise in GSH and GST activities, which quench ROS and OS, protecting cellular lipids from damage.

By strengthening the antioxidant defense mechanisms, these concentrations of DCF may have a protective effect that counters the oxidative damage typically associated with higher concentrations. However, the authors suggest that LPO increases at higher concentrations, eventually reaching the “zone of compensation”. In this zone, the “beneficial low-dose response” is no longer efficient in reducing damage, and biomarker levels are compared to those observed in the control group [120].

Also, McRae et al. [112] observed lower levels of CAT activity in the gills of *Galaxias maculatus* after exposure to DCF. While DCF is commonly linked to increased OS due to higher ROS production and damage to antioxidant enzymes, the impact appears to be tissue-specific and dose-dependent. Notably, the reduction in LPO at the highest exposure concentration indicates that lowered CAT activity may be a sign of some other protective mechanism rather than oxidative defense impairment. Relatively, it may denote a redirection of the protective mechanism toward different forms of antioxidant defenses, possibly the enhancement of SOD or glutathione-related pathways, that reduce oxidative injury.

The ecotoxicological impacts of realistic DCF concentrations remain largely unexplored. Most of the studies used DCF concentrations in the mg/L range, meaning these observations should be interpreted with caution, given the complexity of interactions between chemical substances and fish and the possible health impacts on long-term ecosystems. Nonetheless, chronic or high-dose exposure has been shown to overwhelm these protective barriers, resulting in devastating oxidative injury and physiological damage. The variability in tissue-specific responses and the incomplete recovery of antioxidant systems after exposure underscores the long-term ecological risks associated with DCF pollution in aquatic environments, highlighting the need for further investigation into its sub-lethal and chronic effects.

#### 3.1.2. The Influence of Ibuprofen (IBU) on OS in Various Fish Species

Widely used by humans due to its analgesic, fever-reducing, and anti-inflammatory properties, ibuprofen (IBU) is another commonly detected, non-steroidal anti-inflammatory medicine found in aquatic environments. The living organism’s metabolism of IBU includes hydroxylation and carboxylation or oxidative biotransformation followed by glucuronidation, which results in two principal metabolites, hydroxy-ibuprofen (OH-IBU) and carboxy-ibuprofen (CA-IBU) [122]. Although it is considered biodegradable, IBU has an estimated field half-life of 4 to 32 days [123]. The continued discharge into aquatic ecosystems due to widespread human use leads DCF to be classified as a “pseudo-persistent” compound. The possible ecological effects, particularly for fish exposed to low, yet chronic, concentrations of the IBU, are pretty alarming due to the sustained persistence of IBU.

The concentration of IBU in aquatic environmental samples is typically found in the range of ng/L to μg/L (Table 5). For example, IBU was detected in surface waters of the USA at a concentration of 0.2 µg/L [124], while in Brazil, it ranged from 0.326 to 2.094 µg/L [125]. In Europe, the average concentration of IBU in groundwater is 3 ng/L, with a maximum recorded concentration of 395 ng/L [126].

Studies have shown that IBU can cause genotoxic effects in fish and histopathological lesions in vital tissues like gills and kidneys [130], alter immune functions [131], and have chronic effects, including reduced sperm motility and impaired hatching success [132]. Also, IBU exposure changes OS enzyme activity (Table 6). However, these responses differ by IBU concentration and fish species, with each species having a different degree of detoxification and antioxidant response.

Stancová et al. [133] investigated the effects of IBU exposure in *Tinca tinca* at environmental concentrations of 0.02, 0.2, and 2 μg/L over 35 days. The study reported significant biochemical effects, such as decreased GPx and CAT activity.

These changes may upregulate OS and LPO and downregulate GST activity, a key enzyme in detoxification mechanisms. However, no oxidative damage in lipids was reported despite these biochemical changes. This suggests a possible adaptive response to low-level OS during long-term exposure. Nevertheless, the absence of temporal data from the study on antioxidant enzyme activity and LPO levels limits the understanding of how these parameters evolved, making it difficult to assess potential mitigation mechanisms. The findings indicate that the fish may have developed adaptive strategies to counteract OS throughout the experiment.

The biochemical responses observed in males of *Rhamdia quelen* following IBU exposure suggest a complex interaction between OS and the fish’s antioxidant defense system. The increased GST activity in the posterior kidney across all exposure groups (0.1, 1, and 10 μg/L) indicates an adaptive detoxification response, as GST plays a crucial role in neutralizing ROS and helping in the conjugation of toxic metabolites for excretion.

The higher GPx activity and GSH concentration at the highest exposure concentration (10 μg/L) further support the activation of antioxidant defense mechanisms: GPx catalyzes the reduction in H_2_O_2_ and lipid peroxides, thereby avoiding damage to cells. However, the lack of considerable changes in SOD, CAT, and LPO levels indicates that the OS induced by IBU exposure did not surpass the kidneys’ capacity to maintain redox homeostasis. The authors found no alteration in DNA integrity, further supporting these findings and suggesting that the protective mechanisms successfully prevented the genotoxic effects of IBU.

Likewise, the liver and gills exhibited no significant changes, indicating successful metabolic detoxification and limited oxidative effects in these tissues [114]. This research highlights that the kidney functions as the leading biochemical alteration site after exposure to IBU because it processes and eliminates xenobiotic metabolism and excretion. In contrast, the exposure of *Danio rerio* to environmentally relevant concentrations of IBU (0.1–11 μg/L) led to substantial OS development throughout various tissues, including the brain, gills, liver, and gut. Higher amounts of IBU exposure and higher durations (28 days) cause the antioxidant system to become exhausted until severe oxidative damage occurs [135]. IBU exposure at 11 μg/L resulted in significant biochemical responses, reflecting OS and the activation of antioxidant defense mechanisms. LPO levels were notably increased in the liver at 7, 14, and 28 days, suggesting oxidative damage and lipid peroxidation. In addition, SOD activity was significantly elevated in the liver at 7, 14, and 28 days, as well as in the gut after 14 days, indicating an adaptive antioxidant response. CAT activity also increased significantly in the brain after 21 days, further supporting the role of antioxidant enzymes in countering IBU-induced oxidative stress. Moreover, GPx activity was significantly enhanced in the gills and liver at 21 and 28 days, highlighting the prolonged activation of antioxidative defenses in response to IBU exposure.

Similarly, *Oncorhynchus mykiss* exposed to different concentrations of IBU (2 and 200 μg/kg) by feed ingestion led to notable biochemical changes across different organs. IBU reduced GPx activity in the gills, suggesting weakened antioxidant defense. For instance, IBU increased GR activity and TBARS in the liver, signaling oxidative damage. However, in the posterior kidney, IBU did not change CAT activity, reflecting an antioxidant response improvement. Along with these changes, no DNA damage was observed, indicating that the genomic integrity of the organism was preserved [136].

Altogether, these findings suggest that IBU may trigger OS by enhancing ROS production and enzyme disruption. Still, some fish species might be able to utilize some compensatory defense mechanisms to counteract the damage. Nonetheless, the continuous discharge of IBU in aquatic environments remains a concern for aquatic life and ecosystem homeostasis.

#### 3.1.3. The Influence of Acetylsalicylic Acid (ASA) and Paracetamol (APAP) on OS in Various Fish Species

In addition to NSAIDs such as IBU and DCF, other drugs frequently found in the aquatic environment include acetylsalicylic acid (ASA), commonly known as aspirin, and paracetamol (APAP) or acetaminophen [137,138].

ASA is widely recognized for its anti-inflammatory, analgesic, and antipyretic properties. At the same time, APAP is widely employed worldwide for pain relief and fever reduction, and it is commonly found in sewage treatment plant effluents, surface water, and drinking water.

Recent studies have revealed the presence of ASA in aquatic ecosystems at concentrations ranging from 0.011 to 0.855 μg/L in the North Sea and the Scheldt Estuary [139] and from 0.025 to 0.29 μg/L in the Lis River, Portugal [129]. In Msunduzi River from Africa, ASA appears in higher concentration [140] (Table 7).

Similarly, APAP has been detected at concentrations of 9.6–183 ng/L in Romanian waters [141], 420–610 ng/L in Angke and Ancol, Jakarta Bay, Indonesia [142], and 65 μg/L in the Tyne River, UK [6], as well as in higher concentrations of 246 μg/L in Spanish waters [143].

Although these pollutants are found in aquatic ecosystems at sublethal concentrations, studies have shown that the exposure of fish to ASA can lead to OS and decrease an organism’s ability to detoxify these substances. Gayen et al. [145] exposed *Labeo rohita* to several concentrations of ASA (1, 10, and 100 μg/L) for 7, 14, 21, and 28 days. The study revealed that OS correlated with the dose-dependent and time-dependent decreases in antioxidant enzyme activities (CAT, GPx, and GR) and GSH content. GST activity increased with higher doses but was not influenced by the duration of exposure. Additionally, the authors observed that LPO increased with the dose and time of exposure. Analyzing the presented results, it can be concluded that the concentration and duration of exposure impact these biomarkers significantly, since almost all parameters proved to be significantly affected by the concentration and exposure duration concerning these biomarkers. The decreased activity in SOD and GSH in the liver of *Labeo rohita* suggests changes in the oxidative balance, in addition to damage to the antioxidant defense system from ASA exposure. The decrease in the antioxidant enzymes indicates that fish are more susceptible to severe OS due to a lack of adaptive mechanisms. The exposure of *Mugilogobius abei* at ASA concentrations of 0.5, 5, and 50 μg/ L for 24 h, 72 h, and 168 h showed significant changes in antioxidant and OS markers. There was a general increase in SOD, CAT, GPx, and GST activities, while GSH content showed a decrease after 24 and 72 h of exposure and increased significantly following 168 h. The LPO content increased over the exposure period, indicating OS, although it decreased after 168 h. These results suggest that exposure to ASA does induce OS in organisms, with different responses over time [146] (Table 8).

The exposure of *Danio rerio* to ASA at 4 and 40 μg/L increased the activities of GST, GR, CAT, and GPx, indicating an initial antioxidant response. However, the increase in CAT activity at 4 μg/L was insignificant, indicating a weak enzymatic reaction. LPO levels decreased due to the potential activation of antioxidant defenses, preventing excessive lipid peroxidation [147].

Regarding the impact of environmental concentrations of APAP on fish, the literature findings noted teratogenic-neurotoxic-cardiotoxic effects in the embryos/larvae of *Clarias gariepinus* [148]. Additionally, other studies have established that chronic exposure might significantly alter the histology and function of organs concerned with ion and nutrient homeostasis in rainbow trout [149].

Generally, APAP undergoes a metabolic transformation in the body, resulting in the formation of ROS, primarily due to its conversion to N-acetyl-p-benzoquinone imine (NAPQI), an electrophilic metabolite. NAPQI is known to increase ROS levels, including superoxide anion, hydroxyl radical, and hydrogen peroxide, as documented by Yen et al., 2007 [150].

According to Guilkoski et al. [151], the elevation of ROS levels induced by APAP metabolism is corroborated by the observed induction of SOD activity and protein carbonylation in male fish of *Rhamdia quelen* exposed to environmental concentrations of APAP (0, 0.25, 2.5 μg/L) for 21 days. Notably, GPx activity remained unchanged, suggesting that the H_2_O_2_ produced by SOD could be retained within the cells rather than converted to H_2_O in an efficient manner, which might lead to the protein carbonylation observed in fish after exposure to both low and high concentrations of APAP, as well as DNA damage in hepatocytes exposed only to the lower concentration of the drug.

**Table 8:** OS responses of several fish species exposed to environmental concentrations of ASA and APAP.

Pharmaceutical Product	Species	Concentration and Time of Exposure	Main Findings	Ref.
ASA	*Labeo rohita*	1, 10, 100 μg/L for 7, 14, 21, and 28 days	(-) SOD, CAT, GPx, GRed, and GSH in liver at al conc.; (+) GST and LPO.	[145]
	*Mugilogobius abei*	0.5, 5, and 50 μg/ L for 24, 72, and 168 h	(+) SOD, CAT, GPx, and GST activity; (-) GSH after 24 and 72 h; (+) GSH 168 h; (+) LPO; (-) LPO after 168 h	[146]
	*Danio rerio*	4; 40 μg/ L for 28 days	(+) GST; (+) GRed; (+) CAT; (+) GPx; (-) LPO	[147]
APAP	*Rhamdia quelen*	0, 0.25, and 2.5 µg/ L, for 21 days	(+) SOD activity at a concentration of 2.5 µg/L; (-) GST at all concentrations; No modification of GPx, GSH, and LPO	[151]
	*Rhamdia quelen*	0.25, 2.5, and 25 µg/L	In liver: (+) GSH-; (-) SOD; (-) LPO at 0.25 µg/L; In gonads: (-) GST at 25 µg/L; (+) SOD at 0.25 µg/L; (-) GPx at all conc.	[152]
	*Danio rerio* embryos	150, 300, 450, 600, 750, 900, 1050, and 1200 μg/L	(+) LPO and CAT; (+) SOD from conc. of 300–1200 μg/L	[153]
	*Anguilla anguilla*	5, 25, 125, 625, and 3125 μg/L	In liver: (+) GST at 625 and 3125 mg/L; LPO remained unaltered In gills: (-) GST; (+) LPO	[154]

Note: (+) increase activity; (-) decrease activity.

Similarly, Perussolo et al. 2023 [152], noted alterations in SOD and GPx activity, along with changes in other biomarkers like GST and GSH, indicating a dose-dependent and tissue-specific response to APAP exposure. For instance, GSH levels increased in the liver at 2.5 µg/L, while SOD activity decreased at 0.25 µg/L, resulting in unbalanced antioxidant defenses. Additionally, LPO levels decreased at 0.25 µg/L, suggesting reduced lipid oxidative damage. In the gonads, GST activity declined at 25 µg/L, and SOD activity increased at 0.25 µg/L, possibly as a compensatory response. GPx activity was reduced at all concentrations, signaling OS. These findings underscore that APAP exerts its effect in a tissue-specific and dose-dependent manner on OS, highlighting the complex nature of its mechanism.

In the study of Rosas-Ramírez et al. [153], the effects of APAP on zebrafish embryos were analyzed in different concentrations: 0, 150, 300, 450, 600, 750, 900, 1050, and 1200 μg/L (Table 7). The increased MDA and CAT activity due to APAP exposure (15–1200 μg/L) implicated elevated levels of H_2_O_2_ released during exposure. Additionally, increased SOD activity was reported in *Danio rerio* embryos exposed to APAP at concentrations ranging from 300 to 1200 μg/L. Nunes et al. [154] investigated the effects of APAP exposure on European eel (*Anguilla anguilla*) at concentrations of 5, 25 (reported in aquatic environment [3]), 125, 625, and 3125 μg/L. The authors reported no significant changes in OS at environmentally relevant concentrations (Table 8). However, an unexpected response was observed at higher concentrations, characterized by the reduced activity of CAT and GST alongside increased LPO levels. This indicates compromised cellular oxidative defense and results in elevated oxidative damage. Therefore, it can be deduced that eels possess detoxifying properties to prevent disruption to their metabolism, thus allowing them to maintain primary energy pathways.

In conclusion, it has been proven that the presence of ASA and APAP in aquatic ecosystems induces OS, impairs antioxidant defense, and promotes LPO in fish. Some species induce various adaptive enzymatic mechanisms to counteract oxidative damage, while prolonged exposure eventually leads to more serious cellular and metabolic disorders. Additional studies are needed to understand the extent of ecological risks associated with the chronic exposure of fish to pharmaceuticals.

#### 3.1.4. The Influence of Antibiotics on Fish OS

The widespread use of antibiotics results in the considerable contamination of aquatic environments. Their excessive use poses a significant risk to the health of fish and aquatic ecosystems [155]. Even at low concentrations, antibiotics persist in water and can bioaccumulate in aquatic environments, progressively increasing their levels and being toxic to aquatic organisms [156].

The persistence and bioaccumulation of these substances, even at low levels, heighten the potential risks to aquatic life and add to the growing concern of antibiotic resistance [157,158]. Table 9 presents data on the concentrations of various pharmaceutical pollutants detected in aquatic environments across different locations worldwide. The reported concentrations include azithromycin (2819 ng/L) in the Leça River, Portugal [159], oxytetracycline at two sites (399.5 ng/L in the Danube River, Sulina area and 612 μg/L in the Xiao River, China [160]), and several other antibiotics such as sulfamethazine, sulfamethizole, and trimethoprim, measured in Vietnamese waters, with ranges spanning from 4 ng/L to 328 ng/L [161].

Research generally suggests that the exposure of fish to antibiotics induces a stress response by disrupting their oxidoreductive balance, thereby increasing levels of ROS, and the inactivation process of enzymes like SOD, CAT, and GPx, thus damaging cellular constituents, for example, lipids, proteins, and DNA [162]. Also, long-term exposure can overwhelm these protective mechanisms, compromise physiological functions [163], induce OS, and increase fish disease susceptibility [164]. Generally, these specific changes depend on a few factors, including the type and dosage of the antibiotic, as well as the species and developmental stage of the fish (Table 10).

For instance, a study performed by Almeida et al. [165] observed the long-term (2 months) effects of oxytetracycline (OTC) on zebrafish (*Danio rerio*). In this research, zebrafish were exposed to different concentrations of OTC (0, 0.1, 10, and 10,000 mg/L), with lower concentrations (0.1 and 10 mg/L) reported in environmental waters. The findings showed a significant decrease in GST and CAT enzyme activities (Table 10). This reduction suggests that extended exposure to antibiotics may lower antioxidant defense activity, resulting in OS and tissue damage due to the accumulation of OTC, which can subsequently affect enzyme activity. However, as Massarsky et al. [166] noted, antioxidant enzyme activities might differ in effectiveness depending on OS intensity.

Exposure to sulfamethoxazole (SMZ) has been proven to exert dose-dependent effects on juvenile Nile tilapia (*Oreochromis niloticus*) at concentrations of up to 100 μg/L over 7 and 30 days. Biochemical and molecular markers revealed oxidative stress responses. At low concentrations (1 and 10 μg/L), SMZ enhanced antioxidant enzyme activities (SOD, CAT, and GPx), increased glutathione (GSH) levels, and reduced lipid peroxidation (LPO), suggesting the compensatory activation of defense mechanisms. Oxidative damage was evident at higher concentrations (100 μg/L) through the inhibition of SOD activity, the depletion of GSH, and even more severe LPO. Transcriptional changes in antioxidant enzyme genes corroborate this finding, illustrating the bifunctional nature of SMZ: at low concentrations, the drug would tend to evoke adaptive responses, while at higher doses, it would upset the oxidative balance. The dose-dependent effects reinforce the argument that sub-lethal endpoints deserve increased consideration when assessing the environmental risks of pharmaceutical pollutants [167].

**Table 10:** OS responses of several fish species exposed to environmental concentrations of antibiotics.

Pharmaceutical Product	Species	Concentration and Time of Exposure	Main Findings	Ref.
OTC	*Danio rerio*	0, 0.1, 10, and 10,000 mg/L for 2 months	(-) GST and CAT	[165]
SMZ	*Oreochromis niloticus*	0, 1, 10, and 100 μg/L SMZ for 7 and 30 days	In liver (+) SOD, CAT, GPx, and GSH at 1 and 10 μg/L CAT and GSH, (-) LPO; At 100 μg/L SMZ (-) SOD and GSH; (+) LPO at both 7 and 30 days.	[167]
CIP	*Danio rerio*	0.7 µg/L, 100, 650, 1100, and 3000 µg/L for 28 days	(+) GST at 0,7 and 100 µg/L; (-) GST at 650, 1100, and 3 000 μg/L; (-) GRed at conc. of 1100 and 3000 µg/L; (-) GPX, at all tested concentrations, except for the 100 µg/L; (-) LPO at 100 μg/L	[168]

Note: (+) increase activity; (-) decrease activity.

Ciprofloxacin (CIP) exposure to zebrafish (*Danio rerio*) at an environmental concentration of 0.7 μg/L, and higher concentrations of 100, 650, 1100, and 3 000 μg/L, induced variable responses in antioxidant enzymes and OS markers, even at 0.7 μg/L (Table 10). For GST activity, this significant upregulation paradoxically supports the role of this enzyme in the adaptation response to oxidative challenge. On the other hand, pronounced GRed inhibition was the other effect observed at higher concentrations (1100 and 3000 μg/L). These aspects may suggest disrupting the antioxidant regeneration system under more severe exposure. Similarly, GPx activity was inhibited at all tested concentrations except 100 μg/L, highlighting the concentration-specific effect on enzymatic detoxification pathways.

Notably, the concentration of thiobarbituric acid reactive substances (TBARSs), which measure lipid peroxidation, was only reduced at 100 μg/L, suggesting a possible protective effect at this intermediate level [168]. Worthy of note are the complexities in the impacts of CIP, as they generally seem to follow a concentration gradient, with enzymes displaying adaptive and adverse responses.

#### 3.1.5. The Influence of Antiepileptic Drugs on Fish OS

Of the common antiepileptic drugs within the aquatic environment, carbamazepine (CBZ) is among the most widely detected [169]. CBZ is an antiepileptic drug derived from dibenzoazepine, and it is used as an antiepileptic agent in the treatment of certain types of epilepsy and also for neuropathic pain. Conventional methods at WWTPs do not biodegrade CBZ and it may enter the aquatic environment by different pathways [170]. These risks posed by CBZ, which may be absorbed and bioconcentrated by fish, are significant. CBZ concentrations in aquatic ecosystems have been detected at different levels. Studies report concentrations from 20 to 49 ng/L [171], while in other studies, the concentrations ranged from 0.1 to 1.3 µg/L [172].

The chronic exposure of fish to CBZ has been shown to modulate liver GST activity, whereas CAT activity declines after 63 days of exposure [173]. Furthermore, chronic exposure to CBZ at environmentally relevant concentrations has also been mentioned to cause OS and neurotoxic effects. Studies highlight the impact of CBZ concentrations, even at 1 or 10 µg/L over a 28-day exposure period [174]. These findings indicate that CBZ pollution in aquatic environments causes physiological changes in fish physiology, including OS and probable neurotoxic consequences (Table 11).

In a study conducted by Liang et al. [175], common carp (*Cyprinus carpio*) was exposed to various environmentally relevant subacute concentrations of CBZ (0, 1, 5, 50, and 100 μg/L) for 28 days. The results showed significant changes in the activity of liver enzymes in the antioxidant defense system, indicating high oxidative stress imposed by CBZ. Specifically, during the first seven days of exposure, both enzymes SOD and GRed showed an initial spike in their activities. However, after the 14th day of exposure, the activities of these enzymes had decreased significantly compared to the control group, remaining low until the 28th day of exposure. In contrast, the CAT activity increased throughout the exposure period compared to the control group. Additionally, GST activity showed a significant increase. It is suggested that the decline in SOD and GRed enzymes may be associated with lipid peroxidation, direct OS from ROS, decreased protein levels for ROS scavenging, or energy deficiency (NADPH) resulting from long-term exposure to CBZ [176].

Gasca Perez et al. [177] proved that the acute exposure of common carp to CBZ produces OS effects. Fish were exposed to a concentration of 2000 µg/L CBZ for 12–96 h. Significant changes in the OS markers were recorded by the authors in various organs of the common carp. For instance, LPO levels in the brain showed a notable decrease after 24, 48, and 72 h of exposure, implying that this reduction results from possible methods of adaptation to oxidative damage. SOD activity in the liver, gills, and brain decreased significantly after 12 h of exposure, indicating an early onset of OS induced by CBZ. CAT activity showed a pronounced reduction in the susceptibility of antioxidant defense against OS induced by CBZ in all studied organs during the exposure period. The GPx activity patterns seem to show discrepancy among organs, with an initial increase in the liver at 48 h and then a decline, while reduced GPx activity was observed in gills and brain tissues throughout the exposure time. These findings highlight organ-specific responses to CBZ exposure and emphasize the potential ecological implications of pharmaceutical contaminants in aquatic environments.

**Table 11:** OS responses of several fish species exposed to environmental concentrations of antiepileptic drugs.

Pharmaceutical Product	Species	Concentration and Time of Exposure	Main Findings	Ref.
CBZ	*Cyprinus carpio*	0, 1, 5, 50, or 100 µg/L of CBZ for 28 days	(+) CAT and GRed at 5 and 50 µg/ L) at 100 µg/L (-) GRed.; (-) SOD.	[175]
CBZ	*Cyprinus carpio*	2000 µg/L exposure from 12 to 96 h	(-) LPO in the brain after 24, 48, and 72 h; (-) SOD in liver, gills and brain; (-) CAT; (+) GPx in liver, after 48 h; (-) GPx at 96 h; (-) GPx in the brain and gills.	[177]
CBZ	*Danio rerio*	1; 10 µg/L of CBZ for 28 days	At 1 µg/L: (+) SOD and CAT in the brain; (+) CAT and MDA; (-) SOD in the liver; At 10 µg/L: (+) SOD and CAT in the brain; (+) CAT and MDA in the liver	[174]

Note: (+) increase activity; (-) decrease activity.

#### 3.1.6. The Influence of Antidepressant Drugs on Fish OS

Antidepressant drugs are used in humans suffering from mental health disorders. Studies have shown that these drugs are increasingly detected in aquatic environments because of their long persistence and incomplete removal during wastewater treatment [178,179]. These pharmaceuticals can significantly impact fish by changing their biochemical and physiological processes.

Specifically, antidepressants affect OS biomarkers and the activity of antioxidant defense mechanisms in fish. It is well established that OS can lead to protein oxidation, resulting in altered functionality and new low molecular weight aggregates [180].

Among these drugs, fluoxetine (FLX) is the most frequently detected in aquatic environments, with concentrations ranging from 0.33 to 32.1 ng/L [181] and 0.012 to 1.4 μg/L [159,160]. FLX has been found to induce oxidative stress in various organs of fish, including the liver, gut, brain, and gills. Also, Orozco-Hernández et al. [180] assessed the potential toxicological effects of FLX at environmentally relevant concentrations (5, 16, and 40 ng/L) during a 96 h acute exposure of *Danio rerio.* The authors’ findings demonstrated increased activity levels of SOD, CAT, and GPx in the liver, intestine, brain, and gills. In particular, it was reported that the level of MDA significantly increased in embryos of *Danio rerio* and the brain tissue after 96 h of exposure to FLX at concentrations between 5 and 40 ng/L. This indicates OS in these sensitive developmental and neurological tissues, even at low environmental concentrations of FLX.

Also, other authors have reported elevated values of CAT and SOD enzymatic activity in juvenile *Argyrosomus regius* after 15 days of exposure to FLX concentrations of 300 and 3000 ng/L [182]. In another study, acute exposure for 3 and 6 days to 0.1 μg/L of FLX was reported to increase CAT and SOD activities and the total antioxidant capacity (TAC) in adult *Carassius auratus* livers [183].

In contrast, other studies have reported decreased antioxidant activities following exposure to moderate and high concentrations of fluoxetine. Ding et al. [184] found that SOD activity was inhibited in adult *Carassius auratus* after a 7-day exposure to 4 and 100 μg/L of FLX. Likewise, Cunha et al. [185], reported a significant decrease in the SOD content of *Danio rerio* embryos exposed for 80 h to 0.0015 and 0.5 μM FLX, followed by an increase in CAT activity at the same concentrations (Table 12). A possible explanation for these fluctuations may be attributed to the fact that antioxidant enzymes are influenced by exposure time, which can significantly impact their activity. The increased activity of CAT typically indicates an increased effort to overcome OS. Thus, it can be inferred that the fish face oxidative stressors and the body works to clear ROS. On the other hand, persistently elevated CAT activity could also indicate overworked antioxidant defense that leads to damage in cells over time. Additionally, exposure to FLX increased LPO in the liver of *Carassius auratus* at concentrations of 4, 20, and 100 μg/L after 7 days, indicating oxidative damage. Increased levels of LPO usually indicate the existence of damage caused by OS to lipid membranes. Damage to cellular integrity and function has been seen to make tissues undergo inflammation and, in severe cases, result in cell death. However, if high LPO levels are not accompanied by other signs of OS, such as modifications in antioxidant enzyme activity, this can indicate an altered state of the balance between oxidative damage and repair, which may have long-term consequences for the fish’s health. This could indicate the oxidative effect of FLX on proteins, such that they lose functionality and form new low molecular-weight aggregates. However, low LPO occurred in the juvenile *Argyrosomus regius* exposed for 15 days to 0.3 and 3 μg/L of FLX [182], highlighting that responses differ among species and conditions to which they were exposed.

Nevertheless, the exact mechanism by which FLX induces oxidative stress is not fully understood. It is believed that this pharmaceutical agent triggers OS through multiple pathways. Several researchers have suggested that FLX may affect mitochondrial function. It has been suggested that FLX inhibits the electron transport chain in mitochondria and, more precisely, the activities of complexes I and III, resulting in the leakage of electrons and the overproduction of ROS [186]. Consequently, the disruption of mitochondrial function by FLX can lead to increased ROS production, ultimately resulting in the induction of oxidative stress.

#### 3.1.7. The Influence of Pharmaceutical Mixtures on Fish Oxidative Stress

While laboratory studies typically assess the impact of individual pharmaceutical compounds, real-world scenarios present a more complex picture. In natural environments, fish are exposed to various pharmaceutical substances, leading to a cumulative or synergistic effect, commonly referred to as the “cocktail effect.” This multifactorial exposure significantly impacts fish physiology and behavior in ways that cannot be fully captured by laboratory studies that focus on single toxicants. For instance, the presence of a mixture of NSAIDs at concentrations ranging from a few ng/L to a few μg/L has been frequently reported in marine and estuarine waters [91], surface waters, groundwater [187,188], and even in drinking water [189].

Beyond pharmaceuticals, contaminants such as pesticides, industrial chemicals, and heavy metals frequently coexist in aquatic systems, constituting a more complex mixture of stressors [190,191]. The implication of such mixtures in challenging aquatic ecosystems is that their additive effects always test the hypotheses formulated based on single-species toxicity, considering that an integrated approach is essential for evaluating interference with fish health and OS biomarkers [192].

For example, exposure to more than one NSAID at the same time can overwhelm the detoxification and repair mechanisms of the fish’s body by providing cumulative concentrations of these substances. In the study by Hodkovicova et al. [136], the toxic effects of the oral administration of environmental doses of DCF and IBU and their mixture on rainbow trout (*Oncorhynchus mykiss*) were investigated. After exposing the fish to these NSAIDs for six weeks, the study revealed visible signs of inflammation and OS, along with impaired homeostasis and innate immunity, particularly in fish exposed to the combination of DCF and IBU (2 μg/kg DCF and 2 μg/kg IBU). The reduction in the GPx activity in the gills of fish exposed to the mixture of DCF and IBU indicates that these organs might be experiencing oxidative stress, potentially leading to damage at the cellular or tissue level.

The results showed that common carp (*Cyprinus carpio*) subjected to a mixture of DCF and APAP (50 μg of each/L, 1:1) showed tissue specificity in OS responses, which elucidates the physiological roles and routes of exposures. (Table 13) [193]. An increase in SOD and CAT activities in the brain and gills suggest an adaptive response to elevated ROS. In contrast, higher levels of GPx activity in the brain highlight its susceptibility to oxidative damage. Additionally, the liver exhibits decreased SOD, increased GPx, and LPO, which, taken collectively, are indicative of OS that could be considered significant enough to overwhelm its protective antioxidant mechanism during detoxification processes. The liver and gills exhibited increased LPO, reflecting membrane impairment caused by excessive free radicals and indicating their roles in pollutant detoxification and stress caused by direct environmental exposure to contaminants, respectively. The increase in antioxidant enzyme activity in some tissues counteracts the effects of ROS, while other tissues, especially the liver, show signs of injury that could be a primary target of pharmaceutical toxicity.

While certain pharmaceutical mixtures have been shown to cause significant OS in aquatic organisms, Beghin et al. [192] proved that not all combinations lead to detrimental effects. In their study, juvenile rainbow trout (*Oncorhynchus mykiss*) were exposed to a mixture of five pharmaceuticals (carbamazepine, irbesartan, paracetamol, naproxen, and diclofenac), from the categories of neuroleptic, antihypertensive, analgesic and nonsteroidal anti-inflammatory drugs at concentrations of 1×, 10×, and 100× the median levels found in the Meuse River, Belgium, over 42 days (Table 13). The results showed no significant changes in GST activity, while GSH levels significantly decreased after 24 h, showing a concentration-dependent decline by day 42, with a more significant reduction in the 10× group. GSH depletion correlated with reduced GPx activity, while CAT activity was lower in the 100× group on day 1. Nevertheless, the detoxification mechanisms of fish appeared to have cleared pharmaceuticals, preventing significant oxidative damage.

## 4. Conclusions

This review presents the impact of the most common pharmaceuticals found in the aquatic environment (non-steroidal anti-inflammatory drugs (NSAIDs), antibiotics, antiepileptics, and antidepressant drugs) on the fish OS response.

As a main conclusion, we observed that the research on the effects of pharmaceuticals on aquatic life at ecologically relevant concentrations is limited. Most studies that investigate these pharmaceuticals’ effects on fish tend to focus on high concentrations that are not representative of the low, environmentally relevant levels typically found in natural water systems. While these high-concentration studies provide valuable insights into acute toxicity and potential mechanisms of action, they may overestimate the risks and fail to capture subtle, chronic effects as a result og environmental conditions.

Studies regarding the biomarkers of OS following exposure to environmentally relevant concentration levels of pharmaceuticals are essential for understanding these substances’ subtle and chronic effects on aquatic organisms.

Alterations in the oxidative stress biomarkers of fish exposed to different reported concentrations of environmental pollutants include the increased production of ROS, that is accompanied by significant fluctuations in the activity of some essential antioxidant enzymes like SOD, CAT, LPO, GSH, and GPX. These modifications reflect the physiological stress caused by pharmaceutical compounds and their potential to disturb redox homeostasis. All these changes indicate initial adaptive responses or slight cellular disturbances, unlike high-dose exposures, which only show the mechanisms of acute toxicity. Indeed, under an adaptive cellular or tissue-level change, it may begin to present prior to requiring pathological status, such as an antioxidant enzyme upsurge that indicates alleviation rather than oxidative damage. There are many different non-oxidative pathways through which a lot of drugs can exert damage, and thus, all therapeutic agents cannot be assessed for their toxicological effects using redox biomarkers alone. These changes may be adaptive but can also indicate cellular stress with potential long-term consequences. Biochemical changes that are relatively poorly understood in terms of impacts, especially at low environmentally realistic exposure levels, on fish health, welfare, and ecological functions were not fully established. Assessing the effects of these substances is challenging due to the dynamic variability in their concentrations and ratios, as well as the number of pharmaceuticals involved. Also, a significant gap is that few studies employ multiple biomarkers to evaluate contaminant effects across tissues and systems in fish, an approach that is essential for understanding species-specific toxicity, which rarely varies widely. It is important to understand that OS biomarkers for the detection of biochemical effects have their associated limitations. Individual variability, differences between fish species, and temporal variations in expression complicate data interpretation. The responses may also be transient or compensatory and may not linked to any long-term toxicity or detrimental ecological consequences. An interesting issue here is that while OS biomarkers might serve as early warnings, they should also be well integrated within a larger integrative framework composed of behavioral, physiological, and histopathological endpoints to build stronger toxicity assessments.

Although pharmaceuticals are present in aquatic environments at low levels, they still pose significant threats, especially when they are a part of complex mixtures. These environmental pollutants, including pharmaceutical products, are not found in isolation but consist of active substances and their metabolites. Furthermore, these pharmaceuticals frequently interact with other environmental pollutants, such as microplastics, heavy metals, and industrial chemicals, forming complex mixtures with unpredictable toxic effects. For example, microplastics can serve as carriers for pharmaceuticals and heavy metals, affecting their bioavailability and persistence in aquatic ecosystems. Such interactions could cause synergism or antagonism, which ultimately may lead to issues in fish and ecosystems as a whole. Assessing these pollutant mixtures’ combined toxicity is a significant challenge that requires extensive studies to understand their impact on the environment.

In conclusion, exposing fish to environmental concentrations of pharmaceuticals tends to expose them mainly to chronic, long-term toxic effects rather than acute ones. This prolonged exposure is associated with the increased production of reactive oxygen species (ROS), increased lipid peroxidation production, compromised enzymatic activity, and decreased antioxidant defenses such as glutathione and catalase. Such biochemical derangements suggest that exposure duration and intensity significantly influence organisms’ response.

To address these issues, comprehensive studies are needed to explore the long-term ecotoxicological effects of pharmaceuticals in low concentrations (ng/L). In addition, effective monitoring and assessment would be necessary to prevent possible exposure and harm to aquatic ecosystems. Beyond the biochemical disruptions caused by pharmaceuticals, they affect essential biological functions such as fish growth and reproduction. Changes in OS responses can interfere with endocrine signaling pathways, impair gametogenesis, and impede larval development. Furthermore, prolonged oxidative damage might result in decreased reproductive success, developmental abnormalities, and diminished growth rates, threatening fish populations and biodiversity. These findings highlight the importance of exploring the connections between OS, physiological performance, and population-level effects in fish exposed to environmentally relevant concentrations of pharmaceuticals. Such studies are essential to comprehend and address the broader ecological impacts of pharmaceutical pollution.

Future work should include the refinement of these OS biomarkers and integration with transcriptomics and proteomics to deliver complementary improvements towards understanding possible mechanisms of pharmaceutical-induced toxicity in fish.

## Figures and Tables

**Table 1:** Common pharmaceutical active compounds (PhACs) detected in aquatic environments.

Class of PhACs	Examples	Reference
Antibiotics	Ciprofloxacin, sulfamethoxazole, tetracycline, azithromycin	[15,16,17]
Analgesics and anti-inflammatory drugs	Ibuprofen, diclofenac, aspirin, naproxen	[18,19,20,21]
Antidepressants	Fluoxetine	[18]
Anticonvulsants and mood stabilizers	Carbamazepine	[22,23]
Beta-blockers	Atenolol, propranolol, metoprolol	[24,25]
Hormones	Estradiol, ethinylestradiol, progesterone	[26,27]
Antihistamines	Diphenhydramine	[28]

**Table 2:** Enzymatic antioxidants and their role as biomarkers in oxidative stress (OS) modulation.

Biomarker	Function	Effects	Reference
SOD	Converts superoxide anion (O_2_^−^) into hydrogen peroxide (H_2_O_2_) and O_2_	First-line defense against ROS, prevents oxidative damage and lipid peroxidation	[83]
CAT	Decomposes hydrogen peroxide (H_2_O_2_) into water and oxygen	Protects cells from H_2_O_2_ toxicity, maintains redox homeostasis	[84,85]
GPx	Reduces hydrogen peroxide (H_2_O_2_) and lipid peroxides using glutathione (GSH), producing oxidized glutathione (GSSG)	Detoxifies peroxides and protects lipid membranes from oxidative damage	[86]
GST	Conjugates glutathione (GSH) to xenobiotics and lipid peroxides, aiding detoxification	Neutralizes ROS, supports phase II detoxification, and prevents cellular oxidative damage.	[87]
GRed	Regenerates reduced glutathione (GSH) from oxidized glutathione (GSSG) using NADPH	Maintains glutathione homeostasis, supports antioxidant defenses, and regulates cellular metabolism	[88]

Note: SOD—superoxide dismutase; CAT—catalase; GPx—glutathione peroxidase; GST—glutathione-S-transferase; GRed—glutathione reductase.

**Table 3:** The occurrence of DCF in various environmental waters worldwide.

Environmental Concentration	Sampling Point	Reference
166–252 ng /L	Argeș River, Romania	[18,99]
0.54 μg/L	Central European surface waters	[100]
0.06 to 0.71 µg/L, 0.06 to 0.45 µg/L—DCF; DCF metabolites- 0.07 to 0.42 µg/L for 40-hydroxydiclofenac, 5-hydroxydiclofenac.	Effluents of sewage treatment plants in Germany	[101]
5 to 20 µg/L	Yamuna River, India	[102]
8500 ng/L	Korangi drain, Pakistan	[103]
20 ng/L	Danube River, Romania	[18]
7–90 ng/L	Danube River, Serbia	[104]
435 ng/L	Lake Tegel and Havel River, Germany	[105]
>800 ng/ L	Stream gauge in Mess Basin, Luxemburg	[106]

**Table 4:** OS responses in several fish species exposed to environmental concentrations of DCF.

Species	Concentration and Time of Exposure	Main Findings	Ref.
*Hoplias malabaricus*	0; 0.2; 2.0; 20 μg/kg after intraperitoneal inoculation with 12 doses	(+) SOD, GPx, LPO, and GSH in the liver; (-) GST in the liver; No modifications to CAT activity	[107]
*Danio rerio* (embryos and larvae)	0; 0.5; 5; 50, and 500 μg/L for 96 h	(+) CAT activity and GPx at 500 μg/L; (-) GSTs in all concentrations	[108]
*Oreochromis niloticus*	0; 250; 320; 480 μg/L for 28 days	(+) LPO; (+) GRed, GPx, and GSH; (-) SOD and CAT	[109]
*Rhamdia quelen*	0; 0.2; 2, and 20 μg/L for 21 days	(-) SOD; (+) GSH; (+) GST in all concentrations- in the liver: (-) LPO at 2 and 20 μg/L; (-) CAT at 2 μg/L No modification of GPx activity	[110]
*Rhamdia quelen*	0; 0.2; 2, and 20 μg/L for 96 h	(+) SOD in the kidney at all concentrations No alteration of CAT and GPx; (-) LPO; significant decrease in DNA damage in the kidney at 20 μg/L	[111]
*Galaxias maculatus*	0.17 and 763 μg/L for 96 h	(-) LPO in gills and kidney at 763 μg/L (+) LPO in the liver at 763 μg/L (+) CAT in the liver at 0.17 and 763 μg/L (-) CAT in gills at 0.17 and 763 μg/L	[112]
*Cyprinus carpio*	100 μg/L for 96 h	(-) SOD in liver; (+) SOD in gills; (+) GPx in gills; (+) CAT in brain; no modification of LPO	[113]

Note: (+) increase activity; (-) decrease activity.

**Table 5:** The occurrence of IBU in various environmental waters worldwide.

Environmental Concentration	Sampling Point	Reference
0.2 µg/L	USA surface waters	[124]
0.326–2.094 µg/L	Brasil	[125]
3-395 ng/L	Europe	[126]
50.6 μg/L	WWTP effluent, Spain	[127]
114.9 ng/L	Beijing, China	[128]
3.32–346 ng/L	Danube River	[99]
723 ng/L	Lima River, Portugal	[129]

**Table 6:** OS responses in several fish species exposed to environmental concentrations of IBU.

Species	Concentration and Time of Exposure	Main Findings	Ref.
*Tinca tinca*	0.02–60 μg/L for 35 days	(-) GST in 60 μg/L; (-) GST No modification of CAT activity and LPO	[133]
*Rhamdia quelen*	0.1, 1, and 10 μg/L for 14 days	(+) GST in all groups; (+) GPx and GSH activity at conc. of 10 μg/L; No change of SOD, CAT, and LPO activity in the posterior kidney; No significant changes in OS biomarkers at all concentrations in the gill and liver:	[134]
*Danio rerio*	0.1–11 μg/L for 7, 14, 21, and 28 days	(+) LPO in the liver after 7,14, and 28 days at 11 μg/L (+) SOD activity in the liver at 7, 14, and 28 days and in the gut after 14 days at 11 μg/L; (+) CAT after 21 days in the brain; (+) GPx after 21 and 28 days in the gills and liver at 11 μg/L (+) GPx activity at 21 and 7 days in gills and gut, at 0.1 μg/L	[135]
*Oncorhynchus mykiss*	2 and 200 μg/kg feed	In gills (-) GPX at all concentrations; In liver (+) LPO and GRed at 200 μg/kg feed; No changes in the activity of antioxidant enzymes in the kidney.	[136]

Note: (+) increase activity; (-) decrease activity.

**Table 7:** The occurrence of ASA and APAP in various environmental waters worldwide.

Pharm.	Environmental Concentration	Sampling Point	Reference
ASA	0.011 to 0.855 μg/L	North Sea and the Scheldt Estuary	[139]
	0.025 to 0.29 μg/L	Lis River, Portugal	[129]
	92.8 μg/L	Msunduzi River, Africa	[140]
APAP	9.6–183 ng/	Romania	[141]
	420–610 ng/L	Angke and Ancol, Jakarta Bay, Indonesia	[142]
	65 μg/L	Tyne River, UK	[6]
	246 μg/L	Spania	[143]
	30–1877 μg/L	Midwest Brazil	[144]

**Table 9:** The occurrence of antibiotics in various environmental waters worldwide.

Pharm.	Environmental Concentration	Sampling Point	Reference
Azithromycin	2819 ng/L	Leça river, Portugal	[159]
Oxytetracycline	399.5 ng/L	Danube River—area Sulina	[141]
	612 μg/L	Xiao River, China	[160]
Sulfamethazine Sulfamethizole Trimethoprim	(15–328 ng/L) (20–174 ng/L) (4–7 ng/L)	Vietnamese waters	[161]

**Table 12:** OS responses of several fish species exposed to environmental concentrations of antidepressant drugs.

Pharmaceutical Product	Species	Concentration and Time of Exposure	Main Findings	Ref.
FLX	*Danio rerio*	5, 16, and 40 ng/L for 96 h	(+) SOD, CAT, and GPx in the liver, intestine, brain, and gills. (+) MDA in the brain and tissues after 96 h at a concentration of 5–40 ng/L	[180]
FLX	*Danio rerio*	0.0015, 0.05, 0.1, 0.5, and 0.8 μM for 80 h	(+) CAT (0.0015 and 0.5 μM) (-) SOD (0.0015 and 0.5 μM)	[185]

Note: (+) increase activity; (-) decrease activity.

**Table 13:** OS responses of several fish species exposed to environmental concentrations of pharmaceutical mixtures.

Pharmaceutical Mixtures	Species	Concentration and Time of Exposure	Main Findings	Ref.
DCF + IBU	*Oncorhynchus mykiss*	DCF—2 and 200 μg/kg); IBU—2 and 200 μg/kg. Combination of DCF and IBU—(2 μg/kg DCF + 2 μg/kg; 200 μg/kg IBU).	In gills: (-) GPx activity at IBU 2 and 200 μg kg/kg and the combination of DCF and IBU; In liver: (+) LPO in DCF, and IBU, and DCF conc. of 200 μg/kg (+) GR activity at IBU 200 μg/kg; In the posterior kidney: (+) CAT at DCF 200 μg/kg.	[193]
DCF + APAP	*Cyprinus carpio*	50 μg of each/L, 1:1)	(+) SOD in the brain; (-) SOD in liver and gills; (+) CAT in the brain and gills; (+) GPx in brain and liver; (+) LPO in liver and gills.	[171]
CBZ, irbesartan, APAP, NPX, DCF	*Oncorhynchus mykiss*	Concentrations of 1×, 10×, and 100× the median levels found in the Meuse River, Belgium, over 42 days	No change of GST; (-) GSH after 24 h; (-) GPx and CAT	[172]

Note: (+) increase activity; (-) decrease activity.

## Data Availability

All the data are available from the first author and can be delivered if required.

## References

[1] Caruso G., Corsi I., Wu C., Bergami E., Corami F., Azevedo-Santos V.M. (2024). Plastic pollution in marine and freshwater biota. Water Biol. Secur..

[2] Ortúzar M., Esterhuizen M., Olicón-Hernández D.R., González-López J., Aranda E. (2022). Pharmaceutical pollution in aquatic environments: A concise review of environmental impacts and bioremediation systems. Front. Microb..

[3] Abaineh A., Ejigu D., Atlabachew M., Dejen E., Tilahun G. (2024). Risks of pesticides on aquatic ecosystems and pesticide management effectiveness in Ethiopia. Int. J. Environ. Sci. Technol..

[4] Aziz K.H.H., Mustafa F.S., Omer K.M., Hama S., Hamarawf R.F., Rahman K.O. (2023). Heavy metal pollution in the aquatic environment: Efficient and low-cost removal approaches to eliminate their toxicity: A review. RSC Adv..

[5] Fent K., Weston A.A., Caminada D. (2006). Ecotoxicology of human pharmaceuticals. Aquat. Toxicol..

[6] Roberts P.H., Thomas K.V. (2006). The occurrence of selected pharmaceuticals in wastewater effluent and surface waters of the lower Tyne catchment. Sci. Total Environ..

[7] IQVIA Institute for Human Data Science. https://www.iqvia.com/insights/the-iqvia-institute/reports-and-publications/reports/the-global-use-of-medicines-2023.

[8] Tiwari B., Sellamuthu B., Ouarda Y., Drogui P., Tyagi R.D., Buelna G. (2017). Review on fate and mechanism of removal of pharmaceutical pollutants from wastewater using biological approach. Bioresour. Technol..

[9] Daughton C.G. (2007). Pharmaceuticals in the environment: Sources and their management. Compr. Anal. Chem..

[10] Nikolenko O., Pujades E., Teixid’o M., S’aez C., Jurado A. (2023). Contaminants of emerging concern in the urban aquifers of Barcelona: Do they hamper the use of groundwater?. Chemosphere.

[11] Chonova T., Keck F., Labanowski J., Montuelle B., Rimet F., Bouchez A. (2016). Separate treatment of hospital and urban wastewaters: A real scale comparison of effluents and their effect on microbial communities. Sci. Total Environ..

[12] Wollenberger L., Halling-Sorensen B., Kusk K.O. (2000). Acute and chronic toxicity of veterinary antibiotics to *Daphnia magna*. Chemosphere.

[13] Brausch J.M., Connors K.A., Brooks B.W., Rand G.M. (2012). Human pharmaceuticals in the aquatic environment: A review of recent toxicological studies and considerations for toxicity testing. Rev. Environ. Contam. Toxicol..

[14] Koubová A., Van Nguyen T., Grabicová K., Burkina V., Aydin F.G., Grabic R., Nováková P., Švecová H., Lepič P., Fedorova G. (2022). Metabolome adaptation and oxidative stress response of common carp (*Cyprinus carpio*) to altered water pollution levels. Environ. Pollut..

[15] Kümmerer K. (2009). Antibiotics in the aquatic environment—A review—Part I. Chemosphere.

[16] Mukhtar A., Manzoor M., Gul I., Zafar R., Jamil H.I., Niazi A.K., Muhammad A., Tae J.P., Muhammad A. (2020). Phytotoxicity of different antibiotics to rice and stress alleviation upon application of organic amendments. Chemosphere.

[17] Sosa-Hernández J.E., Rodas-Zuluaga L.I., López-Pacheco I.Y., Melchor-Martínez E.M., Aghalari Z., Limón D.S., Hafiz M.N., Parra-Saldívar R. (2021). Sources of antibiotics pollutants in the aquatic environment under SARS-CoV-2 pandemic situation. Case Stud. Chem. Environ. Eng..

[18] Chițescu C.L., Kaklamanos G., Nicolau A.I., Stolker A.A.M.L. (2015). High sensitive multi-residue analysis of pharmaceuticals and antifungals in surface water using U-HPLC-Q-Exactive Orbitrap HRMS. Application to the Danube River basin on the Romanian territory. Sci. Total Environ..

[19] Das S.A., Karmakar S., Chhaba B., Rout S.K. (2019). Ibuprofen: Its toxic effect on aquatic organisms. J. Exp. Zool. India.

[20] Patel M., Kumar R., Kishor K., Mlsna T., Pittman C.U., Mohan D. (2019). Pharmaceuticals of emerging concern in aquatic systems: Chemistry, occurrence, effects, and removal methods. Chem. Rev..

[21] Placova K., Halfar J., Brozova K., Heviankova S. (2023). Issues of Non-Steroidal Anti-Inflammatory Drugs in Aquatic Environments: A Review Study. Eng. Proc..

[22] Bahlmann A., Weller M.G., Panne U., Schneider R.J. (2009). Monitoring carbamazepine in surface and wastewaters by an immunoassay based on a monoclonal antibody. Anal. Bioanal. Chem..

[23] Baali H., Cosio C. (2022). Effects of carbamazepine in aquatic biota. Environ. Sci. Process. Impacts.

[24] Sumpter J.P., Runnalls T.J., Donnachie R.L., Owen S.F. (2021). A comprehensive aquatic risk assessment of the beta-blocker propranolol, based on the results of over 600 research papers. Sci. Total Environ..

[25] Young R.B., Borch T. (2009). Sources, Presence, Analysis, and Fate of Steroid Sex Hormones in Freshwater Ecosystems—A review. Reproduction.

[26] Grzegorzek M., Wartalska K., Kowalik R. (2024). Occurrence and sources of hormones in water resources—Environmental and health impact. Environ. Sci. Pollut. Res..

[27] Laurenson J.P., Bloom R.A., Page S., Sadrieh N. (2014). Ethinyl estradiol and other human pharmaceutical estrogens in the aquatic environment: A review of recent risk assessment data. AAPS J..

[28] Berninger J.P., Du B., Connors K.A., Eytcheson S.A., Kolkmeier M.A., Prosser K.N., Valenti T.W., Chambliss C.K., Brooks B.W. (2011). Effects of the antihistamine diphenhydramine on selected aquatic organisms. Environ. Toxicol. Chem..

[29] Aich U., Polverino G., Yazdan Parast F., Melo G.C., Tan H., Howells J., Wong B.B. (2024). Long-term effects of widespread pharmaceutical pollution on trade-offs between behavioural, life-history and reproductive traits in fish. J. Anim. Ecol..

[30] Islam P., Hossain M.I., Khatun P., Masud R.I., Tasnim S., Anjum M., Islam M.A. (2024). Steroid hormones in fish, caution for present and future: A review. Toxicol. Rep..

[31] Overturf M.D., Anderson J.C., Pandelides Z., Beyger L., Holdway D.A. (2015). Pharmaceuticals and personal care products: A critical review of the impacts on fish reproduction. Crit. Rev. Toxicol..

[32] Ribas J.L.C., Zampronio A.R., Silva De Assis H.C. (2016). Effects of trophic exposure to diclofenac and dexamethasone on hematological parameters and immune response in freshwater fish. Environ. Toxicol. Chem..

[33] Grabicova K., Grabic R., Fedorova G., Fick J., Cerveny D., Kolarova J., Turek J., Zlabek V., Randak T. (2017). Bioaccumulation of Psychoactive Pharmaceuticals in Fish in an Effluent Dominated Stream. Water Res..

[34] Corcoran J., Winter M.J., Tyler C.R. (2010). Pharmaceuticals in the aquatic environment: A critical review of the evidence for health effects in fish. Crit. Rev. Toxicol..

[35] Nakano T., Hayashi S., Nagamine N. (2018). Effect of Excessive Doses of Oxytetracycline on Stress-Related Biomarker Expression in Coho Salmon. Environ. Sci. Pollut. Res..

[36] Okon E.M., Okocha R.C., Adesina B.T., Ehigie J.O., Alabi O.O., Bolanle A.M., Adedeji O.B. (2022). Antimicrobial resistance in fish and poultry: Public health implications for animal source food production in Nigeria, Egypt, and South Africa. Front. Antibiot..

[37] Directive 2013/39/eu. https://eur-lex.europa.eu/legal-content/RO/TXT/PDF/?uri=CELEX:32013L0039&from=IT.

[38] Kidd K.A., Blanchfield P.J., Mills K.H., Palace V.P., Evans R.E., Lazorchak J.M., Flick R.W. (2007). Collapse of a fish population after exposure to a synthetic estrogen. Proc. Natl. Acad. Sci. USA.

[39] Commission Implementing Decision (EU) 2018/840 of 5 June 2018 Establishing a Watch List of Substances for Union-Wide Monitoring in the Field of Water Policy Pursuant to Directive 2008/105/EC of the European Parliament and of the Council and Repealing Commission Implementing Decision (EU) 2015/495 (Notified Under Document C(2018) 3362). https://eurlex.europa.eu/legal-content/EN/TXT/PDF/?uri=CELEX:32018D0840.

[40] Lahti M., Brozinski J.M., Jylha A., Kronberg L., Oikari A. (2011). Uptake from water, biotransformation, and biliary excretion of pharmaceuticals by rainbow trout. Environ. Toxicol. Chem..

[41] An X., Tao Y., Wu J., Li Z., Li H., Chen S., Pang Y. (2025). Occurrence, toxicity, ecological risk, and remediation of diclofenac in surface water environments: A review with a focus on China. Environ. Toxicol. Chem..

[42] Menashe O., Raizner Y., Kuc M.E., Cohen-Yaniv V., Kaplan A., Mamane H., Avisar D., Kurzbaum E. (2020). Biodegradation of the Endocrine-Disrupting Chemical 17?-Ethynylestradiol (EE2) by *Rhodococcus zopfii* and *Pseudomonas putida* Encapsulated in Small Bioreactor Platform (SBP) Capsules. Appl. Sci..

[43] European Commission, 2020. EUR-Lex—Regulation (EU) 2020/741 of the European Parliament and of the Council of 25 May 2020 on Minimum Requirements for Water Reuse. Official Journal of the European Union L177, 32–54. https://eur-lex.europa.eu/eli/reg/2020/741/oj/eng.

[44] (2022). Eu. https://eur-lex.europa.eu/legal-content/EN/TXT/PDF/?uri=CELEX:32022D1307.

[45] del Carmen Gómez-Regalado M., Martín J., Santos J.L., Aparicio I., Alonso E., Zafra-Gómez A. (2023). Bioaccumulation/bioconcentration of pharmaceutical active compounds in aquatic organisms: Assessment and factors database. Sci. Total Environ..

[46] Van der Oost R., Beyer J., Vermeulen N.P. (2003). Fish bioaccumulation and biomarkers in environmental risk assessment: A review. Environ. Toxicol. Pharmacol..

[47] Zheng C., Liu J., Cai Y., Jing C., Jiang R., Zheng X., Lu G. (2022). Pharmaceutically active compounds in biotic and abiotic media of rivers receiving urban sewage: Concentrations, bioaccumulation and ecological risk Process. Saf. Environ. Prot..

[48] Manjarrés-López D.P., Peña-Herrera J.M., Benejam L., Montemurro N., Pérez S. (2023). Assessment of wastewater-borne pharmaceuticals in tissues and body fluids from riverine fish. Environ. Pollut..

[49] Taslima K., Selly S.L.C., McAndrew B.J., Penman D.J. (2023). Combined estrogen and elevated temperature treatments induce feminization in Nile tilapia, *Oreochromis niloticus*. Aquac. Rep..

[50] Madureira T.V., Rocha M.J., Cruzeiro C., Galante M.H., Monteiro R.A., Rocha E. (2011). The toxicity potential of pharmaceuticals found in the Douro River estuary (Portugal): Assessing impacts on gonadal maturation with a histopathological and stereological study of zebrafish ovary and testis after sub-acute exposures. Aquat. Toxic..

[51] Brodin T., Piovano S., Fick J., Klaminder J., Heynen M., Jonsson M. (2014). Ecological effects of pharmaceuticals in aquatic systems—Impacts through behavioral alterations. Philos. Trans. R. Soc. B Biol. Sci..

[52] Hong X., Zhao G., Zhou Y., Chen R., Li J., Zha J. (2021). Risks to aquatic environments posed by 14 pharmaceuticals as illustrated by their effects on zebrafish behavior. Sci. Total Environ..

[53] Lakshmi S.D., Geetha B.V., Vibha M. (2024). From prescription to pollution: The ecological consequences of NSAIDs in aquatic ecosystems. Toxicol. Rep..

[54] Brodin T., Nordling J., Lagesson A., Klaminder J., Hellström G., Christensen B., Fick J. (2017). Environmental relevant levels of a benzodiazepine (oxazepam) alters important behavioral traits in a common planktivorous fish, (*Rutilus rutilus*). J. Toxicol. Environ. Health Part A.

[55] Dara M., Carbonara P., La Corte C., Parrinello D., Cammarata M., Parisi M.G. (2023). Fish Welfare in Aquaculture: Physiological and Immunological Activities for Diets, Social and Spatial Stress on Mediterranean Aqua Cultured Species. Fishes.

[56] Ogunwole G.A., Abiya S.E., Amaeze N.H., Eze C. (2021). Antioxidant markers in gills, liver and muscle tissue of the African Sharptooth Catfish (*Clarias gariepinus*) exposed to subchronic levels of Ibuprofen and Dibutyl phthalate. Sci. Afr..

[57] Cubbage T.L. (2019). The Effects of Salinity and Pharmaceuticals on the Physiology of the Sheepshead Minnow (*Cyprinodon variegatus*). Ph.D. Thesis.

[58] Tierney K.B., Kennedy C.J., Gobas F., Gledhill M., Sekela M. (2013). Organic Contaminants and Fish. Fish Physiology.

[59] Burkina V., Zlabek V., Zamaratskaia G. (2015). Effects of pharmaceuticals present in aquatic environment on Phase I metabolism in fish. Environ. Toxicol. Pharmacol..

[60] Kolanczyk R.C., Solem L.E., Schmieder P.K., McKim J.M. (2024). A Comparative Study of Phase I and II Hepatic Microsomal Biotransformation of Phenol in Three Species of Salmonidae: Hydroquinone, Catechol, and Phenylglucuronide Formation. Fishes.

[61] Eapen J.V., Thomas S., Antony S., George P., Antony J. (2024). A review of the effects of pharmaceutical pollutants on humans and aquatic ecosystem. Explor. Drug Sci..

[62] Aranda-Rivera A.K., Cruz-Gregorio A., Arancibia-Hernández Y.L., Hernández-Cruz E.Y., Pedraza-Chaverri J. (2022). RONS and Oxidative Stress: An Overview of Basic Concepts. Oxygen.

[63] Juan C.A., Pérez de la Lastra J.M., Plou F.J., Pérez-Lebeña E. (2021). The Chemistry of Reactive Oxygen Species (ROS) Revisited: Outlining Their Role in Biological Macromolecules (DNA, Lipids and Proteins) and Induced Pathologies. Int. J. Mol. Sci..

[64] McGill M.R., Jaeschke H., Kaplowitz N., DeLeve L.D. (2013). Chapter 4—Oxidant Stress, Antioxidant Defense, and Liver Injury. Drug-Induced Liver Disease.

[65] Ayala A., Muñoz M.F., Argüelles S. (2014). Lipid peroxidation: Production, metabolism, and signaling mechanisms of malondialdehyde and 4-hydroxy-2-nonenal. Oxid. Med. Cell. Longev..

[66] Mythri R.B., Vali S., Bharath M.M.S., Dubitzky W., Wolkenhauer O., Cho K.H., Yokota H. (2013). Oxidative Stress, Protein Damage. Encyclopedia of Systems Biology.

[67] Sharma V., Collins L.B., Chen T.H., Herr N., Takeda S., Sun W., Nakamura J. (2016). Oxidative stress at low levels can induce clustered DNA lesions leading to NHEJ mediated mutations. Oncotarget.

[68] Martins S.G., Zilhão R., Thorsteinsdóttir S., Carlos A.R. (2021). Linking Oxidative Stress and DNA Damage to Changes in the Expression of Extracellular Matrix Components. Front. Genet..

[69] Rhee J.S., Yu I.T., Kim B.M., Jeong C.B., Lee K.W., Kim M.J., Lee S.J., Park G.S., Lee J.S. (2013). Copper induces apoptotic cell death through reactive oxygen species-triggered oxidative stress in the intertidal copepod *Tigriopus japonicus*. Aquat. Toxicol..

[70] Awasthi Y., Ratn A., Prasad R., Kumar M., Trivedi S.P. (2018). An in vivo analysis of Cr^6+^ induced biochemical, genotoxicological and transcriptional profiling of genes related to oxidative stress, DNA damage and apoptosis in liver of fish, *Channa punctatus* (Bloch, 1793). Aquat. Toxicol..

[71] Baines C., Meitern R., Kreitsberg R., Fort J., Scharsack J.P., Nogueira P., Sepp T. (2023). Correlations between oxidative DNA damage and formation of hepatic tumours in two flatfish species from contaminated environments. Biol. Lett..

[72] Moore J.L., Rush L.M., Breneman C., Mohideen M.A.P., Cheng K.C. (2006). Zebrafish genomic instability mutants and cancer susceptibility. Genetics.

[73] Rastogi R.P., Kumar A., Tyagi M.B., Sinha R.P. (2010). Molecular mechanisms of ultraviolet radiation-induced DNA damage and repair. J. Nucleic Acids.

[74] Lerebours A., Stentiford G.D., Lyons B.P., Bignell J.P., Derocles S.A., Rotchell J.M. (2014). Genetic alterations and cancer formation in a European flatfish at sites of different contaminant burdens. Environ. Sci. Technol..

[75] Beckwith L.G., Moore J.L., Tsao-Wu G.S., Harshbarger J.C., Heng K.C. (2000). Ethylnitrosourea induces neoplasia in zebrafish (*Danio rerio*). Lab. Investig..

[76] Groff J.M. (2004). Neoplasia in fishes. Vet. Clin. N. Am. Exot. Anim. Pract..

[77] Dale O.B., Tørud B., Kvellestad A., Koppang H.S., Koppang E.O. (2009). From chronic feed-induced intestinal inflammation to adenocarcinoma with metastases in salmonid fish. Cancer Res..

[78] Estevez M., Valesyan S., Jora M., Limbach P.A., Addepalli B. (2021). Oxidative damage to RNA is altered by the presence of interacting proteins or modified nucleosides. Front. Mol. Biosci..

[79] Kong Q., Lin C.L. (2010). Oxidative damage to RNA: Mechanisms, consequences, and diseases. Cell. Mol. Life Sci..

[80] Mustafa S.A., Karieb S.S., Davies S.J., Jha A.N. (2015). Assessment of oxidative damage to DNA, transcriptional expression of key genes, lipid peroxidation and histopathological changes in carp *Cyprinus carpio* L. following exposure to chronic hypoxic and subsequent recovery in normoxic conditions. Mutagenesis.

[81] Khan F., Garg V.K., Singh A.K. (2018). Role of free radicals and certain antioxidants in the management of Huntington’s disease: A review. J. Anal. Pharm. Res..

[82] Olufunmilayo E.O., Gerke-Duncan M.B., Holsinger R.M.D. (2023). Oxidative Stress and Antioxidants in Neurodegenerative Disorders. Antioxidants.

[83] San Juan-Reyes N., Gómez-Oliván L.M., Galar-Martínez M., Vieyra-Reyes P., García-Medina S., Islas-Flores H., Neri-Cruz N. (2013). Effluent from an NSAID-manufacturing plant in Mexico induces oxidative stress on *Cyprinus carpio*. Water Air Soil Pollut..

[84] Ramos A.S., Correia A.T., Antunes S.C., Goncalves F., Nunes B. (2014). Effect of acetaminophen exposure in Oncorhynchus mykiss gills and liver: Detoxification mechanisms, oxidative defence system, and peroxidative damage. Environ. Toxicol. Pharmacol..

[85] Devi G., Harikrishnan R., Paray B.A., Al-Sadoon M.K., Hoseinifar S.H., Balasundaram C. (2019). Effect of symbiotic supplemented diet on innate-adaptive immune response, cytokine gene regulation and antioxidant property in *Labeo rohita* against *Aeromonas hydrophila*. Fish Shellfish Immunol..

[86] Martınez-Alvarez R.M., Morales A.E., Sanz A. (2005). Antioxidant defenses in fish: Biotic and abiotic factors. Rev. Fish Biol. Fish..

[87] Sherratt P.J., Hayes J.D. (2019). Glutathione S-transferases. Enzyme Systems that Metabolize Drugs and Other Xenobiotics.

[88] Waggiallah H., Alzohairy M. (2011). The effect of oxidative stress on human red cells glutathione peroxidase, glutathione reductase level, and prevalence of anemia among diabetics. N. Am. J. Med..

[89] Ji L.L., Yeo D. (2021). Oxidative stress: An evolving definition. Fac. Rev..

[90] Kumari K., Khare A., Dange S. (2014). The applicability of oxidative stress biomarkers in assessing chromium induced toxicity in the fish *Labeo rohita*. Biomed Res. Int..

[91] Blasco J., Trombini C. (2023). Ibuprofen and diclofenac in the marine environment-a critical review of their occurrence and potential risk for invertebrate species. Water Emerg. Contam. Nanoplastics.

[92] Qiu W., Chen J., Li Y., Chen Z., Jiang L., Yang M., Wu M. (2016). Oxidative stress and immune disturbance after long-term exposure to bisphenol A in juvenile common carp (*Cyprinus carpio*). Ecotoxicol. Environ. Saf..

[93] Mulkiewicz E., Wolecki D., Świacka K., Kumirska J., Stepnowski P., Caban M. (2021). Metabolism of non-steroidal anti-inflammatory drugs by non-target wild-living organisms. Sci. Total Environ..

[94] Swiacka K., Michnowska A., Maculewicz J., Caban M., Smolarz K. (2021). Toxic effects of NSAIDs in non-target species: A review from the perspective of the aquatic environment. Environ. Pollut..

[95] Iacopetta D., Ceramella J., Catalano A., Scali E., Scumaci D., Pellegrino M., Aquaro S., Saturnino C., Sinicropi M.S. (2023). Impact of Cytochrome P450 Enzymes on the Phase I Metabolism of Drugs. Appl. Sci..

[96] Bartha B., Huber C., Schröder P. (2014). Uptake and metabolism of diclofenac in Typha latifolia—How plants cope with human pharmaceutical pollution. Plant Sci..

[97] Oviedo-gómez D.G.C., Galar-Martínez M., García-Medina S., Razo-Estrada C., GómezOliván L.M. (2010). Diclofenac-enriched artificial sediment induces oxidative stress in *Hyalella azteca*. Environ. Toxicol. Pharmacol..

[98] Grillo M.P., Hua F., Knutson G.C., Ware A.J., Li C. (2003). Mechanistic studies on the bioactivation of diclofenac: Identification of diclofenac-S-acyl-glutathione in vitro in incubations with rat and human hepatocytes. Chem. Res. Toxicol..

[99] Chiţescu C.L., Ene A., Geana E.-I., Vasile A.M., Ciucure C.T. (2021). Emerging and Persistent Pollutants in the Aquatic Ecosystems of the Lower Danube Basin and North West Black Sea Region—A Review. Appl. Sci..

[100] Hallare A., Kӧhler H., Triebskorn R. (2004). Developmental toxicity and stress protein responses in zebrafish embryos after exposure to diclofenac and its solvent, DMSO. Chemosphere.

[101] Stuelten D., Zuehlke S., Lamshoeft M., Spiteller M. (2008). Occurrence of diclofenac and selected metabolites in sewage effluents. Sci. Total Environ..

[102] Devika Rani H.K., Parimala B. (2024). Biochemical impacts and environmental risks of diclofenac in aquatic ecosystems: A comprehensive review. Int. J. Entomol. Res..

[103] Scheurell M., Franke S., Shah R.M., Hühnerfuss H. (2009). Occurrence of diclofenac and its metabolites in surface water and effluent samples from Karachi, Pakistan. Chemosphere.

[104] Ebele A.J., Abdallah M.A.E., Harrad S. (2017). Pharmaceuticals and personal care products (PPCPs) in the freshwater aquatic environment. Emerg. Contam..

[105] Heberer T., Adam M. (2004). Transport and attenuation of pharmaceutical residues during artificial groundwater replenishment. Environ. Chem..

[106] Banzhaf S., Nödler K., Licha T., Krein A., Scheytt T. (2012). edox-sensitivity and mobility of selected pharmaceutical compounds in a low flow column experiment. Sci. Total Environ..

[107] Guiloski I.C., Ribas J.L.C., da Pereira L.S., Neves A.P.P., Silva de Assis H.C. (2015). Effects of Trophic Exposure to Dexamethasone and Diclofenac in Freshwater Fish. Ecotoxicol. Environ. Saf..

[108] Bio S., Nunes B. (2020). Acute effects of diclofenac on zebrafish: Indications of oxidative effects and damages at environmentally realistic levels of exposure. Environ. Toxicol. Pharmacol..

[109] Eze C.C., Nwamba H.O., Omeje F.U., Anukwu J.U., Okpe M.N., Nwani C.D. (2021). Effects of Diclofenac on the Oxidative Stress Parameters of Freshwater Fish *Oreochromis niloticus*. J. Appl. Life Sci. Int..

[110] Guiloski I.C., Piancini L.D.S., Dagostim A.C., Calado S.L.M., Fávaro L.F., Boschen S.L., Cestari M.M., Cunha C., Assis H.C.S. (2017). Effects of environmentally relevant concentrations of the anti-inflammatory drug diclofenac in freshwater fish *Rhamdia quelen*. Ecotoxicol. Environ. Saf..

[111] Ghelfi A., Ribas J.L.C., Guiloski I.C., Bettim F.L., Piancini L.D.S., Cestari M.M., Silva de Assis H.C. (2016). Evaluation of biochemical, genetic and hematological biomarkers in a commercial catfish Rhamdia quelen exposed to diclofenac. Bull. Environ. Contam. Toxicol..

[112] McRae N.K., Glover C.N., Burket S.R., Brooks B.W., Gaw S. (2018). Acute exposure to an environmentally relevant concentration of diclofenac elicits oxidative stress in the culturally important galaxiid fish *Galaxias maculatus*. Environ. Toxicol. Chem..

[113] Pandey L.K., Bergey E.A., Lyu J., Park J., Choi S., Lee H., Depuydt S., Oh Y.T., Lee S.M., Han T. (2017). The use of diatoms in ecotoxicology and bioassessment: Insights, advances and challenges. Water Res..

[114] Islas-Flores H., Gomez-Olivan L.M., Galar-Martinez M., Colin-Cruz A., Neri-Cruz N., Garcia-Medina S. (2013). Diclofenac-induced oxidative stress in brain, liver, gill, and blood of common carp (*Cyprinus carpio*). Ecotoxicol. Environ. Saf..

[115] Syed M., Skonberg C., Hansen S.H. (2016). Mitochondrial toxicity of diclofenac and its metabolites via inhibition of oxidative phosphorylation (ATP synthesis) in rat liver mitochondria: Possible role in drug-induced liver injury (DILI). Toxicol. In Vitro.

[116] Jung S.H., Lee W., Park S.H., Lee K.Y., Choi Y.J., Choi S., Hong S.S., Lee B.H. (2020). Diclofenac impairs autophagic flux via oxidative stress and lysosomal dysfunction: Implications for hepatotoxicity. Redox Biol..

[117] Justi L.H.Z., da Silva J.F., Santana M.S., Laureano H.A., Pereira M.E., Oliveira C.S., Guiloski I.C. (2025). Non-steroidal anti-inflammatory drugs, and oxidative stress biomarkers in fish: A meta-analytic review. Toxic. Rep..

[118] Diniz M.S., Matos A.P.A., Lourenço J., Castro L., Peres I., Mendonça E., Picado A. (2013). Liver alterations in two freshwater fish species (*Carassius auratus and Danio rerio)* following exposure to different TiO_2_ nanoparticle concentrations. Microsc. Microanal..

[119] Petersen A., Zetterberg M., Sjöstrand J., Pålsson A.Z., Karlsson J.O. (2005). Potential protective effects of NSAIDs/ASA in oxidatively stressed human lens epithelial cells and intact mouse lenses in culture. Ophthalmic Res..

[120] Feito R., Valcárcel Y., Catalá M. (2012). Biomarker assessment of toxicity with miniaturized bioassays: Diclofenac as a case study. Ecotoxicology.

[121] Praskova E., Plhalova L., Chromcova L., Stepanova S., Bedanova I., Blahova J., Hostovsky M., Skoric M., Maršálek P., Voslarova E. (2014). Effects of subchronic exposure of diclofenac on growth, histopathological changes, and oxidative stress in zebrafish (*Danio rerio*). Sci. World J. Ecotoxicol. Environ. Saf..

[122] Oliveira L.L.D., Antunes S.C., Gonçalves F., Rocha O., Nunes B. (2015). Evaluation of ecotoxicological effects of drugs on *Daphnia magna* using different enzymatic biomarkers. Ecotoxicol. Environ. Saf..

[123] Fono L.J., Kolodziej E.P., Sedlak D.L. (2006). Attenuation of wastewater-derived contaminants in an effluent-dominated river. Environ. Sci. Technol..

[124] Cahill J.D., Furlong E.T., Burkhardt M.R., Kolpin D., Anderson L.G. (2004). Determination of pharmaceutical compounds in surface- and ground-water samples by solid-phase extraction and high-performance liquid chromatography-electrospray ionization mass spectrometry. J. Chromatogr..

[125] Pereira C.D.S., Maranho L.A., Cortez F.S., Pusceddu F.H., Santos A.R., Ribeiro D.A., Cesar A., Guimarães L.L. (2016). Occurrence of pharmaceuticals and cocaine in a Brazilian coastal zone. Sci. Total Environ..

[126] Luo Y., Guo W., Ngo H.H., Nghiem L.D., Hai F.I., Zhang J., Liang J., Wang X. (2014). A review on the occurrence of micropollutants in the aquatic environment and their fate and removal during wastewater treatment. Sci. Total Environ..

[127] Martín J., Camacho-Muñoz D., Santos J.L., Aparicio I., Alonso E. (2012). Occurrence of pharmaceutical compounds in wastewater and sludge from wastewater treatment plants: Removal and ecotoxicological impact of wastewater discharges and sludge disposal. J. Hazard. Mater..

[128] Ma R., Qu H., Wang B., Wang F., Yu Y., Yu G. (2019). Simultaneous enantiomeric analysis of non-steroidal anti-inflammatory drugs in environment by chiral LC-MS/MS: A pilot study in Beijing, China. Ecotox. Environ. Saf..

[129] Paíga P., Santos L.H., Amorim C.G., Araújo A.N., Montenegro M.C.B., Pena A., Delerue-Matos C. (2013). Pilot monitoring study of ibuprofen in surface waters of north of Portugal. Environ. Sci. Pollut. Res..

[130] Lonappan L., Brar S.K., Das R.K., Verma M., Surampalli R.Y. (2016). Diclofenac and its transformation products: Environmental occurrence and toxicity—A review. Environ. Int..

[131] Zhang N., Liu X., Pan L., Zhou X., Zhao L., Mou X., Wang X. (2021). Evaluation of ibuprofen contamination in local urban rivers and its effects on immune parameters of juvenile grass carp. Fish Physiol. Biochem..

[132] Han S., Choi K., Kim J., Ji K., Kim S., Ahn B., Yun J., Choi K., Khim J.S., Zhang X. (2010). Endocrine Disruption and Consequences of Chronic Exposure to Ibuprofen in Japanese Medaka (*Oryzias latipes*) and Freshwater Cladocerans *Daphnia magna* and *Moina macrocopa*. Aquat. Toxicol..

[133] Stancova V., Plhalova L., Blahova J., Zivna D., Bartoskova M., Siroka Z., Marsalek P., Svobodova Z. (2017). Effects of the pharmaceutical contaminants ibuprofen, diclofenac, and carbamazepine alone, and in combination, on oxidative stress parameters in earlylife stages of tench (*Tinca tinca*). Vet. Med..

[134] Mathias F.T., Fockink D.H., Disner G.R., Prodocimo V., Ribas J.L.C., Ramos L.P., Cestari M.M., da Silva de Assis H.C. (2010). Effects of low concentrations of ibuprofen on freshwater fish *Rhamdia quelen*. Environ. Toxicol. Pharmacol..

[135] Sánchez-Aceves L., Pérez-Alvarez I., Gómez-Oliván L.M., Islas-Flores H., Barceló D. (2021). Long-term exposure to environmentally relevant concentrations of ibuprofen and aluminum alters oxidative stress status on *Danio rerio*. Comp. Biochem. Physiol. Part C Toxicol. Pharmacol..

[136] Hodkovicova N., Hollerova A., Blahova J., Mikula P., Crhanova M., Karasova D., Franc A., Pavlokova S., Mares J., Postulkova E. (2022). Non-steroidal anti-inflammatory drugs caused an outbreak of inflammation and oxidative stress with changes in the gut microbiota in rainbow trout (*Oncorhynchus mykiss*). Sci. Total Environ..

[137] Wadhah Hassan A.E. (2017). Occurrence of paracetamol in aquatic environments and transformation by microorganisms: A review. Chron. Pharm. Sci..

[138] Satayeva A., Kerim T., Kamal A., Issayev J., Inglezakis V., Kim J., Arkhangelsky E. (2022). Determination of aspirin in municipal wastewaters of Nur-Sultan City, Kazakhstan. IOP Conf. Ser. Earth Environ. Sci..

[139] Wille K., Noppe H., Verheyden K., Vanden Bussche J., De Wulf E., Van Caeter P., Janssen C.R., De Brabander H.F., Vanhaecke L. (2010). Validation and application of an LC-MS/MS method for the simultaneous quantification of 13 pharmaceuticals in seawater. Anal. Bioanal. Chem..

[140] Agunbiade F.O., Moodley B. (2016). Occurrence and distribution pattern of acidic pharmaceuticals in surface water, wastewater, and sediment of the Msunduzi River, Kwazulu-Natal, South Africa. Environ. Toxicol. Chem..

[141] Ilie M., Deák G., Marinescu F., Ghita G., Tociu C., Matei M., Covaliu I., Covaliu M.R., Yusof S.Y. (2020). Detection of Emerging Pollutants Oxytetracycline and Paracetamol and the Potential Aquatic Ecological Risk Associated with their Presence in Surface Waters of the Arges-Vedea, Buzau-Ialomita, Dobrogea-Litoral River Basins in Romania. IOP Conf. Ser. Earth Environ. Sci..

[142] Koagouw W., Arifin Z., Olivier G.W., Ciocan C. (2021). High concentrations of paracetamol in effluent dominated waters of Jakarta Bay, Indonesia. Mar. Pollut. Bull..

[143] Gómez M.J., Bueno M.M., Lacorte S., Fernández-Alba A.R., Agüera A. (2007). Pilot survey monitoring pharmaceuticals and related compounds in a sewage treatment plant located on the Mediterranean coast. Chemosphere.

[144] Américo J.H.P., Isique W.D., Minillo A., Carvalho S.L., Torres N.H. (2012). Drugs in a sewage treatment plant in the Midwest region of Brazil and the risks to water resources. RBRH.

[145] Gayen T., Tripathi A., Kumari U., Mittal S., Mittal A.K. (2023). Ecotoxicological impacts of environmentally relevant concentrations of aspirin in the liver of *Labeo rohita*: Biochemical and histopathological investigation. Chemosphere.

[146] Wang Y., Wang C., Bao S., Nie X. (2020). Responses of the Nrf2/Keap1 signaling pathway in *Mugilogobius abei* (*M. abei*) exposed to environmentally relevant concentration aspirin. Environ. Sci. Pollut. Res. Int..

[147] Zivna D., Plhalova L., Praskova E., Stepanova S., Siroka Z., Sevcikova M., Svobodova Z. (2013). Oxidative stress parameters in fish after subchronic exposure to acetylsalicylic acid. J. Neuroendocrinol..

[148] Erhunmwunse N.O., Tongo I., Ezemonye L.I. (2021). Acute effects of acetaminophen on the developmental, swimming performance and cardiovascular activities of the African catfish embryos/larvae (*Clarias gariepinus*). Ecotoxicol. Environ. Saf..

[149] Choi E., Alsop D., Wilson J.Y. (2018). The effects of chronic acetaminophen exposure on the kidney, gill and liver in rainbow trout (*Oncorhynchus mykiss*). Aquat. Toxicol..

[150] Yen F.L., Wu T.H., Lin L.T., Lin C.C. (2007). Hepatoprotective and antioxidant effects of *Cuscuta chinensis* against acetaminophen-induced hepatotoxicity in rats. J. Ethnopharmacol..

[151] Guiloski I.C., Ribas J.L.C., Piancini L.D.S., Dagostim A.C., Cirio S.M., Fávaro L.F., de Assis H.C.S. (2017). Paracetamol causes endocrine disruption and hepatotoxicity in male fish *Rhamdia quelen* after subchronic exposure. Environ. Toxicol. Pharmacol..

[152] Perussolo M.C., Vicentini M., Lyra L.S., Silva L.Â., Rodrigues M.D.S., Fernandes L.D.S.P., Assis H.C.S.D. (2023). A multibiomarker approach to investigate paracetamol effects in the reproduction regulatory axis of a male Neotropical catfish *Rhamdia quelen*. Acta Limnol. Bras..

[153] Rosas-Ramírez J.R., Orozco-Hernández J.M., Elizalde-Velázquez G.A., Raldúa D., Islas-Flores H., Gómez-Oliván L.M. (2022). Teratogenic effects induced by paracetamol, ciprofloxacin, and their mixture on *Danio rerio* embryos: Oxidative stress implications. Sci. Total Environ..

[154] Nunes B., Verde M.F., Soares A.M. (2015). Biochemical effects of the pharmaceutical drug paracetamol on *Anguilla anguilla*. Environ. Sci. Pollut. Res..

[155] Wan J., Guo P., Peng X., Wen K. (2015). Effect of erythromycin exposure on the growth, antioxidant system and photosynthesis of *Microcystis flosaquae*. J. Hazard. Mater..

[156] Zhao X.L., Li P., Zhang S.Q., He S.W., Xing S.Y., Cao Z.H., Li Z.H. (2021). Effects of environmental norfloxacin concentrations on the intestinal health and function of juvenile common carp and potential risk to humans. Environ. Pollut..

[157] Ljubojević Pelić D., Radosavljević V., Pelić M., Živkov Baloš M., Puvača N., Jug-Dujaković J., Gavrilović A. (2024). Antibiotic Residues in Cultured Fish: Implications for Food Safety and Regulatory Concerns. Fishes.

[158] Zhang L., Kinkelaar D., Huang Y., Li Y., Li X., Wang H.H. (2011). Acquired antibiotic resistance: Are we born with it?. Appl. Environ. Microbiol..

[159] Fernandes M.J., Paíga P., Silva A., Llaguno C.P., Carvalho M., Vázquez F.M., Delerue-Matos C. (2020). Antibiotics and antidepressants occurrence in surface waters and sediments collected in the north of Portugal. Chemosphere.

[160] Li D., Yang M., Hu J., Ren L., Zhang Y., Li K. (2008). Determination and fate of oxytetracycline and related compounds in oxytetracycline production wastewater and the receiving river. Environ. Toxicol. Chem..

[161] Han Q., Zhao S., Zhang X., Wang X., Song C., Wang S. (2020). Distribution, combined pollution and risk assessment of antibiotics in typical marine aquaculture farms surrounding the Yellow Sea, North China. Environ. Int..

[162] Abu-Zahra N.I.S., Atia A.A., Elseify M.M., Soliman S. (2023). Biological and histological changes and DNA damage in *Oreochromis niloticus* exposed to oxytetracycline: A potential amelioratory role of ascorbic acid. Aquacult. Int..

[163] Matthee C., Brown A.R., Lange A., Tyler C.R. (2023). Factors determining the susceptibility of fish to effects of human pharmaceuticals. Environ. Sci. Technol..

[164] Bojarski B., Kot B., Witeska M. (2020). Antibacterials in aquatic environment and their toxicity to fish. J. Pharm..

[165] Almeida A.R., Tacão M., Machado A.L., Golovko O., Zlabek V., Domingues I., Henriques I. (2019). Long-Term Effects of Oxytetracy1cline Exposure in Zebrafish: A Multi-Level Perspective. Chemosphere.

[166] Massarsky A., Kozal J.S., Di Giulio R.T. (2017). Glutathione and zebrafish: Old assays to address a current issue. Chemosphere.

[167] Hu F., Dong F., Yin L., Wang H., Zheng M., Fu S., Zhang W. (2021). Effects of sulfamethoxazole on the growth, oxidative stress and inflammatory response in the liver of juvenile Nile tilapia (*Oreochromis niloticus*). Aquaculture.

[168] Plhalova L., Zivna D., Bartoskova M., Blahova J., Sevcikova M., Skoric M., Svobodova Z. (2014). The effects of subchronic exposure to ciprofloxacin on zebrafish (*Danio rerio*). Neuroendocrinol. Lett..

[169] Salahinejad A., Meuthen D., Attaran A., Chivers D.P., Ferrari M.C. (2023). Effects of common antiepileptic drugs on teleost fishes. Sci. Total Environ..

[170] Tiehm A., Schmidt N., Stieber M., Sacher F., Wolf L., Hoetzl H. (2021). Biodegradation of Pharmaceutical Compounds and Their Occurrence in the Jordan Valley. Water Res. Manag..

[171] Chiţescu C., Ene A., Bahrim G.E., Enachi E. Pharmaceutical residues monitoring in surface water in Romania. Status and concerns. Proceedings of the Environmental Toxicants in Freshwater and Marine Ecosystems in the Black Sea Basin.

[172] Kim Y., Choi K., Jung J.Y., Park S., Kim P.G., Park J. (2007). Aquatic toxicity of acetaminophen, carbamazepine, cimetidine, diltiazem and six major sulfonamides, and their potential ecological risks in Korea. Environ. Int..

[173] Da Silva Santos N., Oliveira R., Lisboa C.A., Mona e Pinto J., Sousa-Moura D., Camargo N.S., Perillo V., Oliveira M., Grisolia C.K., Domingues I. (2018). Chronic Effects of Carbamazepine on Zebrafish: Behavioral, Reproductive and Biochemical Endpoints. Ecotoxicol. Environ. Saf..

[174] Huiting Y., Xiaohong G., Huihui C., Qingfei Z., Zhigang M., Hongmin L., You G., Jinmiao Z. (2022). Effects of Environment-related Concentrations of Carbamazepine on Antioxidant System and Neurotransmitter System of Juvenile Zebrafish. Asian J. Ecotoxicol..

[175] Liang X., Csenki Z., Ivánovics B., Bock I., Csorbai B., Molnár J., Vásárhelyi E., Griffitts J., Ferincz Á., Urbányi B. (2022). Biochemical Marker Assessment of Chronic Carbamazepine Exposure at Environmentally Relevant Concentrations in Juvenile Common Carp (*Cyprinus carpio*). Antioxidants.

[176] Li Z.H., Li P., Rodina M., Randak T. (2010). Effect of human pharmaceutical Carbamazepine on the quality parameters and oxidative stress in common carp (*Cyprinus carpio* L.) spermatozoa. Chemosphere.

[177] Gasca-Pérez E., Galar-Martínez M., García-Medina S., Pérez-Coyotl I.A., Ruiz-Lara K., Cano-Viveros S., Pérez-Pastén Borja R., Gómez-Oliván L.M. (2019). Short-term exposure to carbamazepine causes oxidative stress on common carp (*Cyprinus carpio*). Environ. Toxicol. Pharm..

[178] Moreira D.G., Aires A., de Lourdes Pereira M., Oliveira M. (2022). Levels and effects of antidepressant drugs to aquatic organisms. Comp. Biochem. Physiol. Part C Toxicol. Pharmacol..

[179] Singh A., Saidulu D., Gupta A.K., Kubsad V. (2022). Occurrence and fate of antidepressants in the aquatic environment: Insights into toxicological effects on the aquatic life, analytical methods, and removal techniques. J. Environ. Chem. Eng..

[180] Orozco-Hernández J.M., Elizalde-Velázquez G.A., Gómez-Oliván L.M., Santamaría-González G.O., Rosales-Pérez K.E., García-Medina S., Galar-Martinez M., San Juan-Reyes N. (2023). Acute exposure to fluoxetine leads to oxidative stress and hematological disorder in *Danio rerio* adults. Sci. Total Environ..

[181] Kosma C.I., Nannou C.I., Boti V.I., Albanis T.A. (2019). Psychiatrists and selected metabolites in hospital and urban wastewaters: Occurrence, removal, mass loading, seasonal influence and risk assessment. Sci. Total Environ..

[182] Duarte I.A., Reis-Santos P., Novais S.C., Rato L.D., Lemos M.F., Freitas A., Fonseca V.F. (2020). Depressed, hypertense and sore: Long-term effects of fluoxetine, propranolol and diclofenac exposure in a top predator fish. Sci. Total Environ..

[183] Pan C., Yang M., Xu H., Xu B., Jiang L., Wu M. (2018). Tissue bioconcentration and effects of fluoxetine in zebrafish (*Danio rerio*) and red crucian cap (*Carassius auratus*) after short-term and long-term exposure. Chemosphere.

[184] Ding J., Lu G., Li Y. (2016). Interactive effects of selected pharmaceutical mixtures on bioaccumulation and biochemical status in crucian carp (*Carassius auratus*). Chemosphere.

[185] Cunha V., Rodrigues P., Santos M.M., Moradas-Ferreira P., Ferreira M. (2016). *Danio rerio* embryos on Prozac—Effects on the detoxification mechanism and embryo development. Aquat. Toxicol..

[186] de Oliveira M.R. (2016). Fluoxetine and the mitochondria: A review of the toxicological aspects. Toxicol. Lett..

[187] Sathishkumar P., Meena R.A.A., Palanisami T., Ashokkumar V., Palvannan T., Gu F.L. (2020). Occurrence, interactive effects and ecological risk of diclofenac in environmental compartments and biota—A review. Sci. Total Environ..

[188] Jurado A., Labad F., Scheiber L., Criollo R., Nikolenko O., Pérez S., Ginebreda A. (2022). Occurrence of pharmaceuticals and risk assessment in urban groundwater. Adv. Geosci..

[189] Sharma J., Joshi M., Bhatnagar A., Chaurasia A.K., Nigam S. (2022). Pharmaceutical residues: One of the significant problems in achieving ‘clean water for all’ and its solution. Environ. Res..

[190] Lushchak V.I. (2016). Contaminant-induced oxidative stress in fish: A mechanistic approach. Fish Physiol. Biochem..

[191] Stoliar O.B., Lushchak V.I. (2012). Environmental pollution and oxidative stress in fish. Oxidative Stress—Environmental Induction and Dietary Antioxidants.

[192] Beghin M., Schmitz M., Betoulle S., Palluel O., Baekelandt S., Mandiki S.N., Kestemont P. (2021). Integrated multi-biomarker responses of juvenile rainbow trout (*Oncorhynchus mykiss*) to an environmentally relevant pharmaceutical mixture. Ecotoxicol. Environ. Saf..

[193] Nava-’Alvarez R., Razo-Estrada A., García-Medina S., G’omez-Olivan L., Galar-Martinez M. (2014). Oxidative stress induced by mixture of diclofenac and acetaminophen on common carp (*Cyprinus carpio*). Water Air Soil Pollut..

